# Geometric principles underlying the proliferation of a model cell system

**DOI:** 10.1038/s41467-020-17988-7

**Published:** 2020-08-18

**Authors:** Ling Juan Wu, Seoungjun Lee, Sungshic Park, Lucy E. Eland, Anil Wipat, Séamus Holden, Jeff Errington

**Affiliations:** 1 Centre for Bacterial Cell Biology, Biosciences Institute, Medical School, Newcastle University, Richardson Road, Newcastle upon Tyne, NE2 4AX UK; 2 Interdisciplinary Computing and Complex BioSystems research group, School of Computing, Newcastle University, Newcastle upon Tyne, NE4 5TG UK; 3 Present Address: Maurice Wohl Clinical Neuroscience Institute, Institute of Psychiatry, Psychology and Neuroscience, King’s College London, London, SE5 9RX UK

**Keywords:** Cell growth, Cellular microbiology

## Abstract

Many bacteria can form wall-deficient variants, or L-forms, that divide by a simple mechanism that does not require the FtsZ-based cell division machinery. Here, we use microfluidic systems to probe the growth, chromosome cycle and division mechanism of *Bacillus subtilis* L-forms. We find that forcing cells into a narrow linear configuration greatly improves the efficiency of cell growth and chromosome segregation. This reinforces the view that L-form division is driven by an excess accumulation of surface area over volume. Cell geometry also plays a dominant role in controlling the relative positions and movement of segregating chromosomes. Furthermore, the presence of the nucleoid appears to influence division both via a cell volume effect and by nucleoid occlusion, even in the absence of FtsZ. Our results emphasise the importance of geometric effects for a range of crucial cell functions, and are of relevance for efforts to develop artificial or minimal cell systems.

## Abstract (web summary)

Bacteria can form wall-deficient variants, or L-forms, that divide by a simple mechanism that does not require the FtsZ-based cell division machinery. Here, Wu et al. study L-forms in microfluidic systems to show the importance of geometric effects for cell growth, chromosome segregation and cell division.

## Introduction

The cell wall is an ancient and highly conserved structure that is almost ubiquitous in the bacterial domain^1^. It provides a tough, elastic, protective outer layer around the cell and is largely responsible for the characteristic shapes associated with different forms of bacteria^2,3^. The wall is the target for many effective antibiotics, and fragments of the wall are recognised by innate immune receptors^4^. Its most critical general role lies in osmoregulation, enabling bacterial cells in dilute environments to withstand the turgor pressure generated by the high osmolarity of the cytoplasm^5^. A large number (~30) of normally essential genes are required for synthesis of the material of the wall, and its spatial regulation during cell growth and division^6,7^.

In the light of the multiplicity of important functions for the wall it is surprising that under certain conditions (isotonic to avoid osmotic lysis) many bacteria, both Gram-positive and Gram-negative, that normally have a cell wall, can thrive in a wall-less state, called the L-form^8,9^. Although L-forms can probably inhabit a range of specialised niches in the environment, they have mainly been studied in the context of their possible role in various chronic diseases and recurrent infections^9–11^.

In previous work with the Gram-positive bacterium *Bacillus subtilis* we have shown that L-form growth requires two types of mutations: one that leads to excess membrane synthesis, and one that counteracts the increased cellular levels of reactive oxygen species (ROS) that occur for reasons that are not fully understood in L-forms^12–14^. Upregulation of membrane synthesis can be achieved directly with mutations affecting the regulation of fatty acid synthesis, or indirectly by inhibiting peptidoglycan precursor synthesis^12^. In some bacteria, inhibiting peptidoglycan precursor synthesis alone seems sufficient to enable L-form growth^15^. While walled bacteria generally divide by a well-regulated binary fission process, division of L-forms of *B. subtilis* and several other bacteria investigated, occurs through a range of poorly regulated and seemingly haphazard events including membrane blebbing, tubulation, vesiculation and fission. Crucially, these division events occur independent of the normally essential FtsZ-based division machine^9,12,16,17^. Our current model for L-form proliferation assumes that division is driven simply by an imbalance between volume and surface area. Support for this idea comes from the fact that we have been unable to identify mutations in genes required for division, other than those that upregulate membrane synthesis^12^. Furthermore, there is a sound mathematical basis for the process^18^ and it has even been replicated in vitro with simple lipid vesicle systems^19^. The simplicity of this division process has led to suggestions that L-form division may be a good model for studying how primordial cells proliferated before the invention of “modern” protein based division machines^1,16,20,21^. It is also of interest as the basis for proliferation in simplified or artificial cell systems^22–24^.

Detailed analysis of L-form proliferation has been hampered by the lack of effective systems for following their growth and division by time-lapse imaging. The cells tend not to remain in focus in liquid culture and attempts to tether them to surfaces can cause flattening and lysis. Thus, many questions about their cell cycle remain unresolved, particularly the extent to which chromosome replication and segregation can be controlled and coordinated with growth and division in cells with pleomorphic shape and no cell wall. (Note that in this paper because many of the cells observed are not undergoing division, we use the term “segregation” for sister chromosomes that have visibly separated, whether or not a division septum separates them).

Here, we report that the use of microfluidic devices that force L-forms into an elongated shape, with cross section similar to that of walled cells, strikingly improves the rate of growth and the efficiency and fidelity of chromosome segregation and other cell cycle processes. The cross-section also influences the rate of division in channels. Despite the lack of requirement for FtsZ, division is strongly biased to internucleoid spaces, as in walled cells. Our results also support the notion of a key role for changes in surface area to volume underlying L-form division. Overall, these results show that simple geometric effects can have a profound impact on the efficiency of fundamental cell cycle processes including growth, chromosome segregation and cell division. They also lend support to the idea that simple biophysical effects such as phase separation and entropic de-mixing (e.g., refs. ^25,26^) underlie key steps in the cell cycle of modern bacteria. The results and methods developed here provide important insights into fundamental principles of cell growth, proliferation and chromosome inheritance and have implications for the development of simplified or artificial cell systems.

## Results

### Irregular division and chromosome segregation in unconstrained L-form cells

**Fig. 1:**
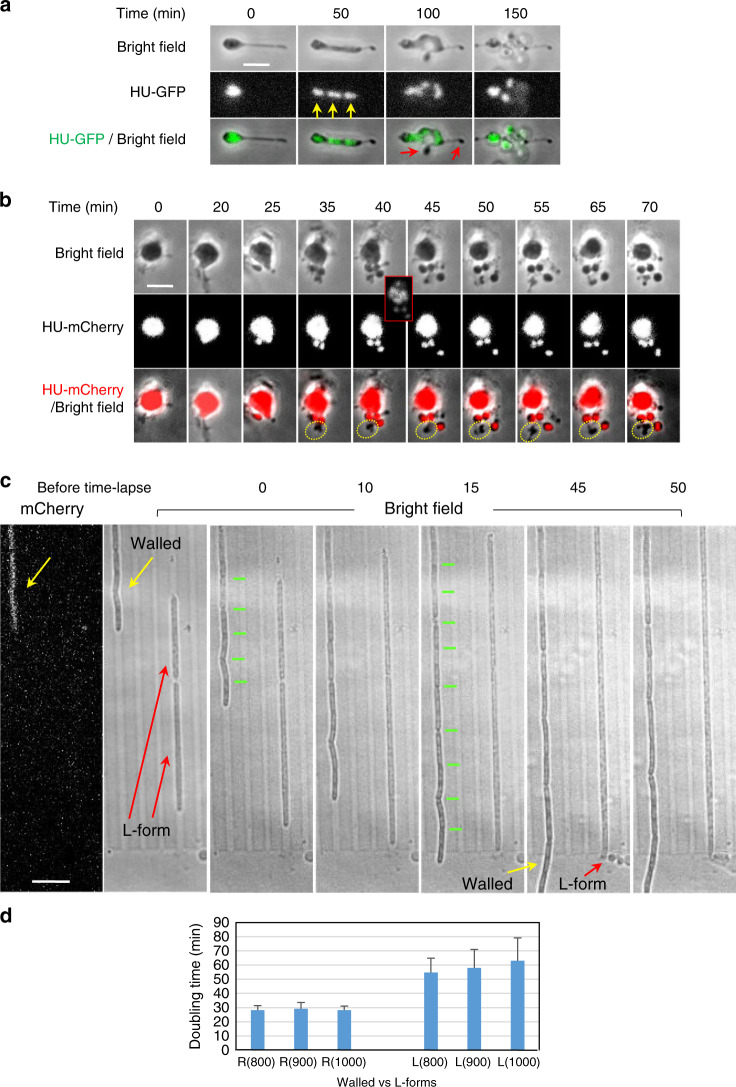
Unconstrained L-form growth in liquid and in microfluidic devices. **a** L-forms growing unrestrained in liquid medium. The figure shows still images from time-lapse microscopy (from Supplementary Movie 1) of L-form cells of strain 4740 (LR2 *Pspac-dnaA ΩamyE*::*neo hbsU-gfp*) growing in a glass bottomed dish at 30 °C. Yellow arrows point to discrete nucleoids and red arrows point to cells devoid of DNA. Min, minutes. Scale bar, 5 µm. This experiment and those presented in (**b**) and (**c**) were all performed more than three times independently, with multiple positions imaged in each experiment, with similar results. **b** L-form cells of strain 4745 (RM121 *ΩamyE*::*hbsU-mCherry rpoC-gfp)* growing in the gutter of a microfluidic system. Example of a series of still images from a time-lapse experiment in which the cells stayed relatively in focus (Supplementary Movie 2). A daughter bleb with no chromosome is circled in yellow. For both **a** and **b** each panel shows bright field images on top, chromosomal DNA labelled with HU-mCherry in the middle, and a merge of the two (DNA in gre en in (**a**) and red in (**b**)) at the bottom. The brightness of the HU-mCherry images (nucleoids) in (**b**) was enhanced so that DNA in smaller cells could be detected. The insert shows an unsaturated image of the cells at t40 min. The time points (min) of the selected still images from the time-lapse experiment are shown above the images. Scale bar, 5 µm. **c** Growth of walled (strain 4742; LR2 *ΩamyE*::*neo hbsU-gfp aprE::P*_*rpsD*_*-mcherry spc ∆xylR::tet*) and L-form (strain 4739; LR2 *ΩamyE*::*neo hbsU-gfp*) *B. subtilis* in microfluidic channels. The pair of images to the left were taken before the time-lapse imaging, showing the mCherry signal in the walled cells on the left (yellow arrows), and two L-form cells that do not express mCherry on the right (red arrows). Short green lines mark division sites. The time points of the selected still images from the time-lapse experiment (5-min intervals) are shown above each image. Scale bar, 5 µm. **d** Growth rates, shown as doubling time (min), of walled and L-form strains in channels of different widths. R: walled cells; L: L-forms. Channel widths (nm) are indicated in brackets. Data are presented as mean values ± SD (standard deviation). Exponentially growing cells from three independent experiments were analysed. For walled cells, 70, 75 and 130 cells were analysed for the 800, 900 and 1000 nm channels, respectively. For L-forms, 179, 199 and 218 cells were analysed for the 800, 900 and 1000 nm channels, respectively. R800 (*n* = 70), R900 (*n* = 75), R1000 (*n* = 68), L800 (*n* = 179), R900 (*n* = 199), R1000 (*n* = 218). See the Methods for more details.

Previous work on *B. subtilis* “primary” L-forms (i.e., L-forms derived directly from walled cells, requiring only one or two mutations), as well as many earlier papers with long-propagated “stable” L-forms, have highlighted the inefficient and rather haphazard mode of proliferation in liquid culture^12,16,17,27^. Fine details of the multiplication process have been difficult to obtain because various methods normally used to fix the position of cells during time-lapse imaging either damage or distort the shape of L-forms, or fail to keep progeny cells in focus. Figure 1a and Supplementary Movie 1 show typical examples of L-form cells (strain 4740) growing unconstrained in liquid medium in a glass-bottomed microscope dish. Cells clearly underwent growth and division but segments of cell mass frequently moved in and out of focus (top and bottom panels of Fig. 1a), making long-term tracking of cells difficult. Note that in this kind of common event, the main cell body appeared to be attached to the glass surface by a fine tube of membrane material which filled up with cytoplasm and DNA as the cell grew. The presence of these fine tubes of membrane has been described in previous L-form publications (e.g., ^16,17^), although their nature and biological significance is unclear.

A key objective of the current work was to characterise the extent to which chromosome replication and segregation remain coordinated with division, so in these and subsequent experiments, chromosomes were labelled with fluorescent fusions to the HU protein, which binds DNA almost non-specifically^28^.

The middle and lower panels of Fig. 1a (and Supplementary Movie 1) show: (i) that discrete nucleoid-like structures could be discerned within L-forms (yellow arrows) but brighter structures, containing either overlapping multiple nucleoids or un-resolved multiple chromosomes, could be seen frequently (e.g., 0 min); (ii) that these could resolve into multiple discrete structures (e.g., arrows at 50 min); and (iii) that the arrangement in larger L-form clusters was complex and difficult to track because of focus and overlapping problems (e.g., 150 min). It also appeared that some cell lobes might be devoid of DNA (e.g., phase dark objects with no associated fluorescence at 100 min (red arrows). (Note that in this and some subsequent figures the fluorescence image brightness was enhanced to enable visualisation of small amounts of DNA. Raw images are available on request).

### Use of microfluidics to constrain L-form movement during growth

Agarose based microfluidic devices offered a possible way to constrain the cells without damaging them, while maintaining them within a focal plane. We fabricated microfluidic devices based on those described by Moffitt et al.^29^ (see ref. ^30^; Supplementary Fig. 1). Each device contained sub-micron-scale linear tracks (channels), imprinted into agarose. The channels were restricted in height (~1.6 µm) to impose a strong z-axis control over the cells as they grew. The growth channels were open, at either one or both ends, to gutters through which growth medium flowed, delivering fresh nutrients.

It turned out that the gutters provided an improved way to image L-form growth without physical constraint. Figure 1b and Supplementary Movie 2 show an example of a common division event within a gutter. Here, a large L-form cell underwent “blebbing” to generate multiple small daughter units, all in fairly good focus. Three of the blebs displayed HU fluorescence in frames from 40 to 70 min, whereas one (circled in yellow in the bottom panels) was non-fluorescent and presumably anucleate. The variation in the sites of division and in the number of nucleoids in daughter cells in these and many other similar experiments showed that chromosome segregation in unconstrained L-forms is poorly regulated and relatively disorganised.

### Imposition of an elongated architecture regularises L-form growth

Surprisingly, when L-forms were trapped in the channelled area of the microfluidic chamber, so that growth would be forced to occur along a fixed longitudinal axis, a strikingly different pattern of growth was observed. Now, the cells grew rapidly and with uniform appearance along the channel (red arrows in Fig. 1c). In the experiment shown, L-forms were mixed with mCherry labelled walled cells (yellow arrows) to enable comparison of their behaviour. The L-forms were almost indistinguishable from the walled cells except that the latter had regular constrictions (due to cell division; indicated by green bars) and a slightly less regular cylindrical shape, perhaps because of frictional drag against the channel walls. However, upon exiting the channels, the difference between walled cells, which continued to grow in straight lines out into the gutter, and the L-forms, which immediately formed chains and clusters of spherical blebs, was striking.

Interestingly, when growing in these channels, the L-forms rarely divided (see the section below). In the typical example shown, despite having similar length increase after 15 min of growth in the channels, clear constrictions corresponding to division sites (marked with short green bars) increased from 5 to at least 9 in the chain of walled cells, but none were evident in the L-forms (Fig. 1c, 15 min).

The microfluidic channel designs in these initial experiments were of two types, featuring repeating patterns of widths approximately 800, 900 and 1000 nm wide, or 600, 700 and 800 nm wide. Walled cells of wild-type *B. subtilis* are approximately 850 nm in diameter^31^, so the channels roughly mimic walled cell dimensions. Under these conditions the growth rate of the L-forms could be readily estimated from the increase in length over time, assuming that the cross-sectional area of the channel and thus of the cell was constant. As summarised in Fig. 1d, in 800 nm channels the average length doubling time of the L-form strain (strain 4739) grown at 32 °C was about 2× of that of the isogenic walled cells (strain SL004) (55 min ± 10.1 vs. 28 min ± 3.3, respectively), a significant improvement compared to the 6–8 h doubling time (optical density) when L-forms were grown unconstrained in liquid culture^16^.

As expected, growth in the wider channels did not alter the width of walled cells (which normally maintain a constant width irrespective of growth rate^31^), nor did it affect their length doubling time (Fig. 1d). The L-forms, however, showed increased length doubling time as the channel width increased (5% for 900 nm and 15% for 1000 nm, respectively).

### Effects of channel width on L-form growth and division

**Fig. 2:**
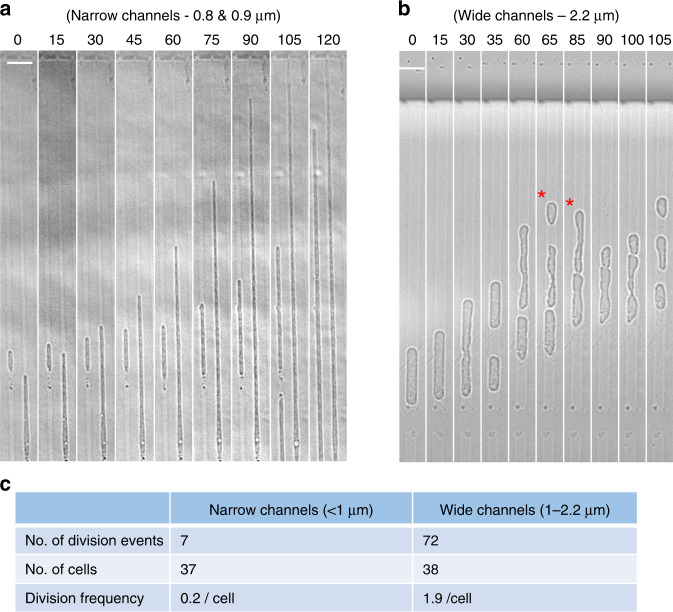
Effect of channel width on L-form division. **a** Lack of division in narrow microfluidic channels. Selected still bright field images of a time-lapse experiment. L-form cells of strain 4739 (LR2 *ΩamyE*::*neo hbsU-gfp*) were loaded into microfluidic chamber (Chip No. 2; channel widths 0.8, 0.9 and 1.0 µm) and grown at 32 °C. Images were captured every 5 min. The small cell on the left was in a 0.9 µm channel; the longer cell on the right was in a 0.8 µm channel. Full set of still images from this time-lapse series is shown in Supplementary Fig. 2. Scale bar, 5 µm. The experiment was performed more than three times independently, and multiple positions were imaged in each experiment, with similar results. **b** In wide channels cell division occurred more frequently. Selected still bright field frames from a time-lapse experiment. The cell shown was in the 2.2 µm wide channel (Chip No. 7). Red stars label cells that escaped from the channel. Strain: 4739 (LR2 *ΩamyE*::*neo hbsU-gfp*). Scale bar, 5 µm. The experiment was performed independently twice, and multiple positions were imaged in each experiment, with similar results. **c** Division frequency of L-forms grown in narrow vs wide channels. Only ‘in-channel’ division events occurred in the first 5 h of the time-lapse experiment were scored. Images from two different experiments were analysed for each channel width [Tabulated data].

The low frequency of division of L-forms trapped in the narrow channels (Figs. 1c and 2a; Supplementary Fig. 2) was unexpected. We previously reported experiments suggesting that L-form division is driven by excess membrane synthesis, creating a high surface area to volume (A/V) ratio that is incompatible with a spherical shape and thus drives shape changes leading to division^12^. Cylindrical shapes have a higher A/V ratio than spheres of the same volume. It was therefore possible that the narrow channels imposed a geometry with high enough A/V to eliminate the driving force for division that occurs in unconstrained (roughly spherical) L-forms. If so, increasing the channel width, and therefore reducing the imposed A/V, might re-enable division. To test this we designed two microfluidic chips with wider channels (Chip No. 6 = 1, 1.2 and 1.4 µm; Chip No. 7 = 1.8, 2.0 and 2.2 µm). As predicted, ‘in-channel’ division occurred much more frequently in these wider channels (e.g., 35, 65, 90 and 105 min frames in Fig. 2b). It needs to be mentioned that the wide channels were only half the length of those of the narrow channels, and so would effectively give only half the chance of observing ‘in-channel’ division in the same time frame, making direct comparisons difficult. Supplementary Figure 3a and Supplementary Movie 3 show a typical example of a long L-form growth sequence in wide channels. Accurate quantitation of division frequency was problematical for several reasons. First, tracking of cells was limited by the channel length, because undivided cells often ‘bubbled’ out of the ends of the channel and this material then disappeared (Fig. 1c, 45 and 50 min frames; and 105 min frame onwards in Fig. 2a), so measurement of total cell length per division was not possible. Second, after division in the wider channels some progeny cells spontaneously escaped from the channels (e.g., cells labelled with a red star in Fig. 2b frames 65 and 85 min, and the 195 min frame in Supplementary Fig. 3a; Supplementary Movie 3) so that, again, their subsequent fate could not be recorded. Finally, an element of stochasticity seemed to arise due to small irregularities in the channels, probably either casting irregularities or debris/thin membrane fibres from the growing L-form cells. Nevertheless, we estimated the difference in division frequency by counting ‘in-channel’ division events in continuous cell lineages over 5 h time courses for channels of different widths. Clustered division events that occurred occasionally in cells with chromosome segregation defects (see below) were excluded from this analysis. The results confirmed that division was rare in narrow (<1 µm) channels (7 division events in 37 cells during the whole time course) and much more frequent in the wider (1–2.2 µm) channels (72 division events in 38 cell lineages) (Fig. 2c). The lower frequency of division in the narrow channels compared with the wide channels is consistent with the A/V model for division in L-forms (see “Discussion”).

### Efficient chromosome segregation in channel constrained L-forms

**Fig. 3:**
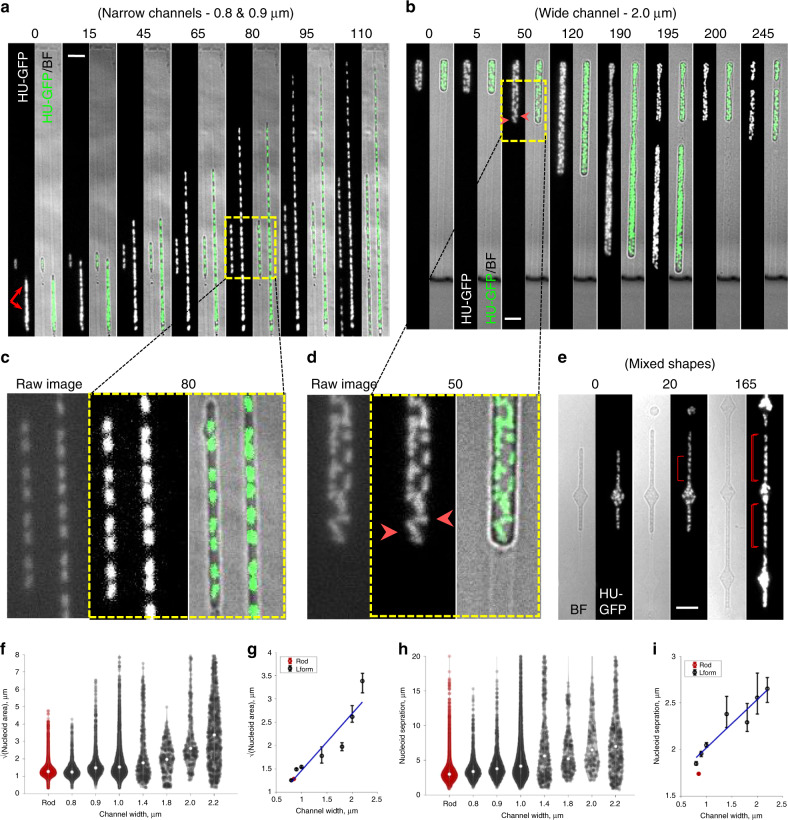
Effect of channel width on nucleoid appearance and arrangement in L-forms. **a**, **c** Regular chromosome segregation in narrow microfluidic channels. Selected frames from the same time-lapse experiment shown in Fig. 2a. For each time frame (min) two images are presented: a HU-GFP image showing the nucleoids (left) and the merge of the GFP image (green) and the corresponding bright field image (grey) (right). The small cell on the left was in a 0.9 µm channel; the large cell on the right was in a 0.8 µm channel. Full set of still images is shown in Supplementary Fig. 2. Yellow boxed region is enlarged and shown in Fig. 3c. An un-processed GFP image at *t*80 min is shown to the left in Fig. 3c. Scale bar, 5 µm. **b**, **d** Irregular chromosome arrangement and distribution in wide channels. Selected frames from Supplementary Movie 4. The cell shown was in a 2.0 µm channel (Chip No. 7). Arrowheads: chromosomes lying horizontally or perpendicularly. Strain: 4739 (LR2 *ΩamyE*::*neo hbsU-gfp*). Yellow boxed region is enlarged and shown in Fig. 3d. An un-processed GFP image at t50 min is shown to the left in Fig. 3d. Scale bar, 5 µm. More time frames are shown in Supplementary Figure 3a. **e** Chromosome arrangement in Chip No. 33, which contained alternating narrow channels and diamond shapes. These are selected frames from Supplementary Movie 6, showing a bright field image on the left and chromosomal DNA (HU-GFP) on the right. Strain: 4741 (LR2 *ΩamyE*::*neo hbsU-gfp aprE::P*_*rpsD*_*-mcherry spc)*. Brackets indicate regions where chromosomes appear regularly distributed. Scale bars, 5 µm. More time frames are shown in Supplementary Fig. 3c. Each experiment was performed at least twice independently, and imaged at multiple positions, with similar results. **f**–**i** Quantitative analysis shows that spatial confinement of L-forms can reproduce near-native nucleoid segregation. **f** Nucleoid size, as measured by the square root of nucleoid area, for walled cells (Rod, red) and L-forms in different channel widths (black). **g** Scatter plot of square root of nucleoid area versus channel width for walled cells (red) and L-forms (black). **h** Nucleoid separation for walled cells (Rod, red) and L-forms in different channel widths (black). **i** Scatter plot of nucleoid separation vs. channel width. Blue line in **g** and **i**: linear fit to the L-form data. For walled cells (red) in **g** and **i**, the “channel width” was set to 850 nm to match the known cell width. Violin plots: Circles indicate the median. Scatter plots: Circles indicate the median, error bars indicated 95% confidence interval from bootstrapping. *n* = 17,766 time-lapse observations of nucleoids descended from 45 mother cells in separate agarose channels. Source data are provided as a Source Data file.

We then examined the effects of channel confinement on nucleoid arrangement and segregation, using HU-GFP fluorescence imaging. When the cells were initially placed in narrow channels, the multiple nucleoids often appeared as large overlapping or un-resolved masses (Fig. 3a, red arrows at 0 min; cells in 0.8 and 0.9 µm wide channels) but as the cells increased in length, these masses gradually resolved into smaller, individual nucleoids (e.g., 80 min).

After this initial phase of resolution many cells showed a remarkably regular pattern of chromosome replication and segregation. For example, in Fig. 3a (full sequence in Supplementary Fig. 2), over a time frame of 110 min, the short cell on the left, containing one nucleoid at 0 min, undertook three sequential successful duplications (times 15, 65 and 110 min), to give 2, 4 and then 8 segregated nucleoids, while the large cell on the right also showed increasingly regular nucleoid arrangement (times 80 and 95 min) (enlarged section shown in Fig. 3c). Supplementary Movie 4 shows another example of large DNA masses resolving into smaller and often regularly spaced nucleoids. Several L-form strains with different genetic origins were tested (including strains 4739, 4741 and 4744; Supplementary Table 1) and all were able to resolve large nucleoids and then regularly distribute the chromosomes when grown in narrow channels. Quantitative analysis of various nucleoid parameters (area, width, eccentricity and internucleoid separation; Fig. 3f–i; Supplementary Fig. 3d, e), showed that, except for eccentricity (see below), L-form nucleoids in 0.8 or 0.9 µm channels appeared remarkably similar to those of walled cells.

These results demonstrate that cell wall synthesis is not required for regular chromosome segregation, at least not when cells are forced to grow under these geometric constraints. Importantly, these findings also definitively exclude any models for chromosome replication or segregation that require pre-existing markers in the cell wall.

### Effects of cell geometry on chromosome segregation

We then examined the effects of channel width on chromosome replication and segregation. Unlike the narrow channels, chromosome arrangement was increasingly perturbed in the wider L-form cells. Stills of typical frames are shown in Fig. 3b, with more examples shown in Supplementary Fig. 3a, b; Supplementary Movies 3 and 5. A close up of the typical nucleoid appearance in a wide channel is shown in Fig. 3d. Although nucleoid lobes similar in size and fluorescence intensity to the individual nucleoids of cells in the narrow channels were evident, they tended to form clumps that split up only infrequently. Inspection of the movies revealed highly dynamic patterns of splitting and coalescence that will merit further investigation. Quantitative measurements of various nucleoid parameters relative to channel width are shown in Fig. 3f–i, and Supplementary Fig. 3d, e. Nucleoid area and nucleoid separation (centroid to centroid) both increased in parallel with increasing channel width, due to the failure of nucleoids to separate efficiently in wider channels. The failure of nucleoid lobes to separate was also manifested in a decrease in nucleoid eccentricity (ratio of nucleoid width to length; Supplementary Fig. 3d) and an increase in nucleoid width, which increased proportional to channel width (Supplementary Fig. 3e).

In support of the close connection between cell width and nucleoid configuration, we noticed that when cells grown in wider channels occasionally became slightly constricted, length wise, perhaps because of damage or miscasting of the agarose, nucleoid separation was strikingly improved (red brackets in Supplementary Fig. 3b; Supplementary Movie 5). To test this further we designed a microfluidic chip with narrow channels interrupted by wider diamond shapes (Fig. 3e, Supplementary Figs. 1 and 3c; Supplementary Movie 6). Nucleoids were well distributed in the narrow (700 nm) part of the channels (red brackets in Fig. 3e; Supplementary Fig. 3c; Supplementary Movie 6). However, on growing into the larger diamond regions, nucleoids lost their regular linear arrangement and spread out in different orientations to fill the space (compare the orientations of the two nucleoids labelled by yellow arrows in Supplementary Fig. 3c, 30 min).

All of these observations and measurements are consistent with the idea that efficient chromosome segregation is dependent on the geometry of the cell and, as is evident from the line plots in Fig. 3g, i, that artificially setting the width of the L-form at about that of walled cells (~850 nm) generates a normal pattern of segregation.

### Division of L-forms mainly occurs between nucleoids

**Fig. 4:**
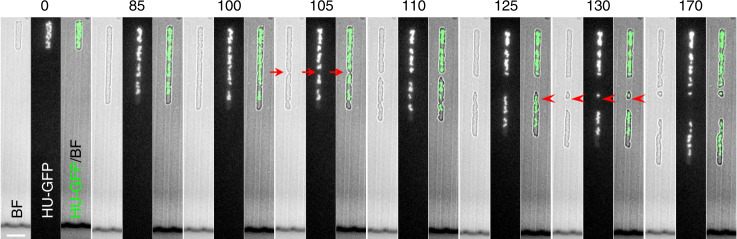
Bisection of chromosomes occurs occasionally in L-forms. A small cell with little DNA (arrowheads in Frame 130 min) appeared to be not growing, possibly because its chromosome is incomplete. These are selected still frames from Supplementarty Movie 7. The cell shown was in a 1.8 µm wide channel (Chip No. 7). Arrows point to division between nucleoids; arrowheads point to possible division through nucleoids. Scale bar, 5 µm. Similar events could be observed occasionally at different locations from independent experiments (repeated at least twice).

**Fig. 5:**
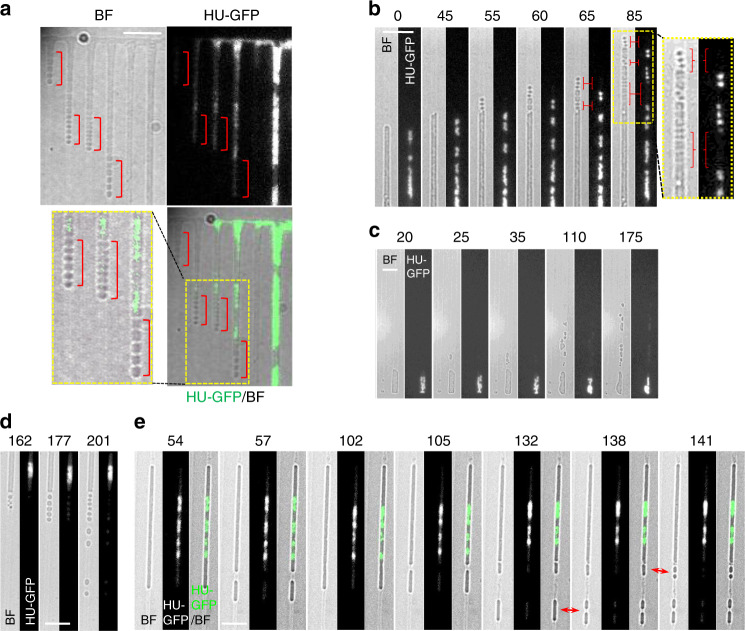
DNA deficiency leads to DNA-free pearling division in channel-confined L-forms. **a**, **b** DNA-less ‘beads’ produced by L-forms in narrow channels under normal growth conditions. Scale bars, 5 µm. Brackets: DNA-free, bead-like cells. **a** Cells in the gutter grew into the narrow channels (0.8–1.0 µm) with the mass of the nucleoid excluded from entry, generating strings of DNA-less beads in the channels. A bright field image is shown on the top left, chromosomal DNA labelled with HU-GFP on the top right, and a merge of the two on the bottom right (GFP in green). Yellow boxed region is enlarged and shown at the bottom left. These are selected still frames from Supplementary Movie 11. Strain: 4739 (LR2 *ΩamyE*::*neo hbsU-gfp*). **b** Small DNA-less cells in narrow channels. Each time frame (min) shows bright field images on the left and chromosomal DNA labelled with HU-GFP on the right. Yellow boxed region is enlarged and shown on the right. Strain: 4742 (LR2 *ΩamyE*::*neo hbsU-gfp aprE::P*_*rpsD*_*-mCherry spc)*. **c** A cell, appeared to be defective in chromosome replication/segregation (for unknown reason), produced many small DNA-free daughter cells of various sizes. These are selected still frames from Supplementary Movie 10. The cell shown was in a 2.2 µm wide channel (Chip No. 7). Each time frame (min) shows bright field images on the left and chromosomal DNA labelled with HU-mCherry on the right. Strain: 4739 (LR2 *ΩamyE*::*neo hbsU-gfp*). Scale bar, 5 µm. **d**, **e** Examples of L-forms inhibited for DNA replication generating regular pearling (**d**) or large DNA-free cells (**e**) in the DNA deficient regions in narrow channels. L-forms of strain 4739 (LR2 *ΩamyE*::*neo hbsU-gfp)* were grown in the presence of the DNA replication inhibitor HB-EmAu in liquid culture and after introduction into a microfluidic device. These are selected still frames from Supplementary Movies 11 (for **d**) and 12 (for **e**). Red arrows in **e** shows anucleate cells dividing. Scale bars, 5 µm. The experiment in (**c**) was performed twice and the other experiments were performed more than three times, independently, with similar results.

Walled bacterial cells segregate sister chromosomes at cell division with high fidelity. The coordination between segregation and division is thought to rely heavily on an effect called nucleoid occlusion. As first described it was proposed to rely on a phase separation between DNA and cytoplasm, together with a tendency of membrane invagination to be impaired in the nucleoid zone^32–34^. More recently nucleoid occlusion proteins, Noc in *B. subtilis*^35^ and SlmA in *E. coli*^36^, were identified that are associated with the chromosome and act to inhibit assembly or constriction of the FtsZ machine in its vicinity. Nevertheless, mutants deficient in these proteins still tend to divide between nucleoids under normal conditions^35,37^. Given that L-form division occurs independently of FtsZ it was interesting to examine whether L-form division is also subject to a nucleoid occlusion effect. Division through the nucleoid is barely detectable in walled cells^38^. Perhaps surprisingly, bisection of nucleoids was also infrequent in L-forms growing in channels. Of 45 division events (excluding the ‘abnormal’ division events that generated anucleate daughter cells—see below) only 4 (9%) appeared to have occurred through a chromosome (e.g., arrowheads in Fig. 4; Supplementary Fig. 4; Supplementary Movies 7 and 8). Thus, although the frequency of bisection was much higher than in walled cells using the FtsZ-based division machine, a large majority of division events (91%) still occurred between nucleoids (e.g., arrows in Fig. 5a, 105 min).

### Division frequency of L-forms is increased by DNA deficiency

We previously postulated that the blebbing or extrusion division events of L-forms could be driven by active nucleoid segregation followed by membrane sealing around the nucleoid^16^. This class of model gains support from in vitro experiments showing that encapsulated nanoparticles or macromolecules can drive tubular extrusions or budding transformations in simple lipid vesicles^39,40^. However, in the channel experiments evidence against this idea arose in rare microfluidic ‘accidents’ of which an example is shown in Fig. 5a (full sequence in Supplementary Movie 9). Material spilling over from the filled channel to the right sequentially entered the adjacent channels leftwards. This material appeared to be deficient in DNA presumably because the chromosome entering the channel was incomplete, damaged or delayed. The precise nature of the defect was unclear but it resulted in a striking series of repeated divisions adjacent to the edge of the visible DNA, giving a string of small spherical compartments (red brackets). Figure 5b shows another example in which multiple small anucleate spheres were generated (‘pearling’; see ‘Discussion’) at the end of a cell and in an unusually large internucleoid region (red brackets in enlarged inset). Similar events occurred in wide channels: the cell in the typical example shown in Fig. 5c (Supplementary Movie 10) appeared to be defective in chromosome replication (no significant change in DNA fluorescence between 0 and 175 min). Multiple irregular sized anucleate blebs were shed from the cell along the channel.

The above effects appeared to occur generally in cells that were deficient in DNA. To test this idea we set up experiments in which L-forms grown in narrow channels were treated with specific inhibitors of DNA synthesis 6(p-hydroxyphenylazo)-uracil (HPUra)^41^ or N3-hydroxybutyl 6-(3-ethyl-4-methylanilino) uracil (HB-EMAU)^42^. The two inhibitors gave similar results, generating elongated cells with few nucleoids, as expected. Importantly, division events were now frequently detected (Fig. 5d, e; Supplementary Movies 11 and 12), even in the narrow channels that do not normally support efficient division (80 out of 93 cells divided). Again this always occurred away from regions occupied by a nucleoid. Many cells exhibited pearling (e.g., panel D and Supplementary Movie 11) but other events were also frequently seen, such as division of the anucleate cells/membrane tubes (Fig. 5e, red arrows; Supplementary Movie 12).

These results appear to exclude the idea that the nucleoid can act positively to promote division and indeed suggest rather that the nucleoid has a negative effect on the division of tubular L-forms.

### Rapid equilibration of nucleoid positioning after asymmetrical cell division

**Fig. 6:**
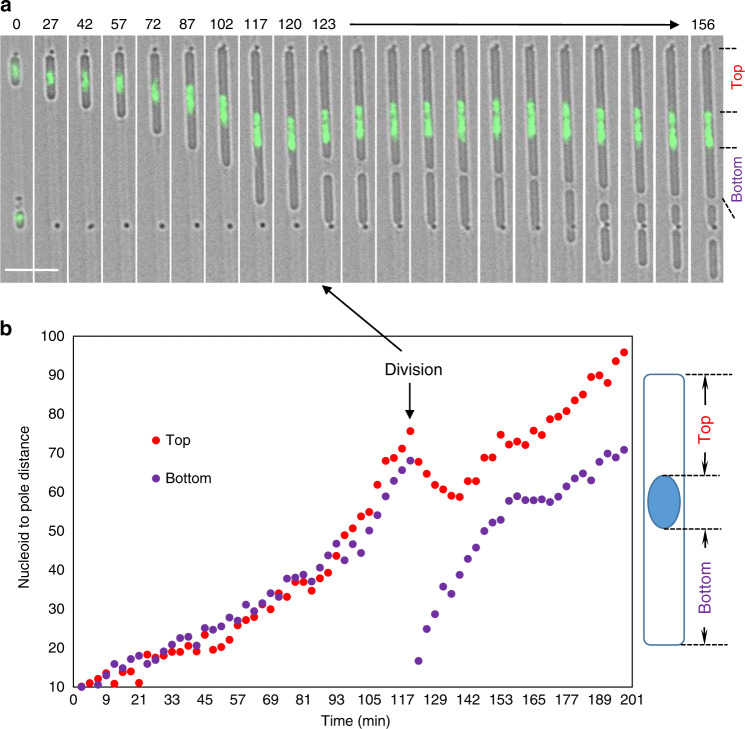
Re-centring of the single nucleoid after asymmetric division in narrow channels. **a** Selected still images from a time-lapse experiment also shown in Supplementary Movie 13, showing a single-nucleate cell growing and dividing in the narrow channel. The merge of the bright field image (grey) and the green fluorescence image of the chromosomal DNA labelled with HU-GFP (green) is shown. After division the asymmetrically located nucleoid moved towards the distal pole to re-centre itself. Such events, which occurred in similar experiments, could be clearly observed only in large cells that had a single nucleoid and divided near the nucleoid, and had a sufficiently long time gap before the second division occurred. More examples are shown in Supplementary Fig. 5. L-forms of strain 4739 (LR2 *ΩamyE*::*neo hbsU-gfp)* were grown in the presence of the DNA replication inhibitor HB-EmAu in liquid culture and after introduction into a microfluidic device. Scale bar, 5 µm. **b** Distances of the nucleoid to the two cell poles over a 2.5 h time course. The distance (arbitrary unit) between the polar edge of the nucleoid to the nearest pole was measured, as explained in the cartoon on the right. Division that occurred at the 123 min frame is marked by arrows.

Wu et al.^26^ showed that single nucleoids in non-dividing *E. coli* cells are robustly positioned at mid-cell, whereas in cells with two nucleoids, they self-organise at one-fourth and three-fourth positions, regardless of the length of the cell. In our experiments with inhibitors of DNA replication, we generated similar elongated cells with mainly centrally located single nucleoids (see earlier time frames in Fig. 6b and Supplementary Fig. 5b, c). Some of these cells carried out an asymmetric cell division event generating one anucleate cell and a cell with a sub-polar nucleoid (Fig. 6a, 123 min, and another example in Supplementary Fig. 5b, c). Interestingly, we noticed that in these cells the nucleoid abruptly changed its direction of movement after division, going back towards the distal cell pole to restore an approximately central position (Fig. 6; Supplementary Fig. 5; Supplementary Movie 13). The initial movement occurred more rapidly than cell elongation, being visible within the first 3 min time frame after division was observed. It is not immediately clear whether this movement can be explained by entropic forces as in the models described by Wu et al.^26^.

## Discussion

There is abundant literature on L-forms (reviewed by refs. ^8,9^), including papers describing L-forms’ irregular almost haphazard mode of proliferation (e.g., refs. ^16,17,27,43^). Parent and daughter L-form cells vary greatly in terms of their size, in contrast to the relatively tight approximately twofold variation in the size of most walled bacteria. Division is also quite difficult to define because cells can form blebs or tubes that can retract and re-fuse with the parent cell^16,17^. However, here we show that by forcing L-forms into an elongated configuration similar to that of walled cells, growth and chromosome segregation are greatly improved. This suggests that geometry plays an important role in cell fitness. Interestingly, Hussain et al.^44^ also observed improved growth for mutant walled *B. subtilis* cells when transitioned from spherical to rod shape, achieved by adjusting the expression level of *tagO* (involved in wall teichoic acid synthesis) and Mg^2+^ concentration, and proposed that lower doubling time of rods is likely due to cell shape and not another effect. Our results with L-forms clearly exclude the involvement of the cell wall. It thus seems that cell function is tightly connected to geometry because parameters such as surface area to volume ratio, cytoplasm to nucleoid, DNA to protein, membrane to cytoplasm, etc, are all directly affected by cell geometry.

Why should an axial organisation, as imposed by the channel or a cylindrical wall be a preferred state? The subject has been reviewed in detail by Young^45^. Obvious possibilities include the following. First, a higher A/V ratio (i.e., a rod shape) can improve nutrient uptake by providing more surface area through which diffusion can occur, and/or more receptors for uptake of specific nutrients. Second, elongation with constant perimeter (i.e., in a cylinder) provides a way to balance the rates of synthesis of cytoplasm and surface: each increment in length (*l*) results in a requirement for 2*π.r.l* in surface area and *π.r*^2^*.l* in volume. If the radius (*r*) is constant, as would normally be the case, cell surface area is directly proportional to volume, irrespective of length. Third, the geometry provides cells with an axis of polarity along which the segregation of chromosomes can occur.

L-form proliferation has recently been identified as an interesting paradigm for how primitive cells could have proliferated before the invention of the cell wall, and as an interesting starting point for the development of artificial or minimal cell systems^1,16,20,22–24^. Genetic experiments showed that various mutations enabling the proliferation of *B. subtilis* L-forms all had in common an upregulation of cell membrane synthesis^12^, leading to the suggestion that excess surface area synthesis could drive proliferation. Theoretical considerations backed up by simple in vitro systems have demonstrated how an increase in surface area at constant volume can drive simple membrane vesicles to divide (e.g., refs. ^19,46^). In rod shaped bacteria, cell surface area (*A*) increases almost proportionately to cell volume (*V*). However, in spherical cells the *A*/*V* ratio decreases during growth. It is not clear what happens to surface area regulation when normally rod-shaped bacteria transition to a non-rod shape, e.g., when the *mreB* system is impaired or the cell wall is lost. Bendezu and de Boer^47^ showed that *E. coli* cells transiting to a spherical (but walled) mutant state accumulate intracellular vesicles that presumably accommodate excess surface material, indicating that *E. coli* tends not to downregulate surface synthesis under these circumstances. However, *B. subtilis* seems to be able to regulate membrane synthesis, as spherical (*rod*) mutants do not seem to generate intracellular vesicles and, as mentioned above, *B. subtilis* L-forms require upregulation of membrane synthesis to proliferate^12^.

Although it will clearly be interesting to follow up with a more detailed quantitative analysis, it is apparent that the narrow channels, which impose a higher A/V ratio on the L-forms, almost eliminate division, whereas the wider channels allow division to occur frequently. These observations are consistent with our previous model for L-form proliferation^12^ in which division is driven by the rate of surface growth exceeding that of volume increase. Shape changes and ultimately the formation of several smaller progeny from one large L-form dissipate the excess surface area generated during growth.

Chromosome spacing and orientation were strikingly improved by the confinement of L-forms in narrow channels. An important conclusion from these experiments is that they exclude any models for chromosome segregation that invoke an essential requirement for specific interactions with the cell wall or cell poles.

Several well conserved proteins have been implicated in chromosome organisation and or movement. The ParAB proteins play a reasonably well defined role in segregation of low copy number plasmids, and homologues are found in the chromosomes of most bacteria. Work on *Caulobacter* and sporulating cells of *B. subtilis*^48–54^ has revealed that these proteins have an active role in movement of origin regions towards cell poles. However, *Caulobacter* is unusual in having highly specialised cell poles and work here and in filamentous *B. subtilis* suggests that poles are not important outside of sporulation. The SMC or MukBEF protein complexes are also found in virtually all bacteria and appear to work by helping to self-condense chromosomes, inhibiting the formation of tangles. However, how sister chromosomes come to occupy different spaces and ultimately move away from each other remains poorly understood in bacteria. Much recent work has focused on the role of entropic forces to drive segregation^25,55–58^. Dekker and colleagues have recently shown that chromosome size, configuration and positions are markedly influenced by the geometry of confinement in non-dividing walled cells perturbed in various ways^26,59^. Our finding that narrow rod-like geometric confinement of L-forms is sufficient to generate robust chromosome segregation strongly supports entropic models of segregation.

Wu and colleagues have recently argued that chromosome size, configuration and position are strongly driven by phase partitioning of the chromosome and cytoplasmic crowders within a defined cellular geometry^26,59^. The behaviour of L-form nucleoids in our channel experiments appears largely consistent with their findings. However, in our experiments, nucleoids re-centred remarkably rapidly after division. Further investigation will be required to determine whether current models of phase partitioning dependent nucleoid positioning are sufficient to explain such rapid re-centring.

Several theoretical and practical papers have highlighted the possible role of macromolecules or nanoparticles in promoting the division of cells or vesicles (e.g., refs. ^39,40^). Based on these ideas it seemed possible that segregating nucleoids could drive the proliferation of L-forms by acting as nanoparticles. This would have an important knock-on effect in that it would ensure that progeny L-form cells contain at least one chromosome. However, our channel experiments showed that involvement of the nucleoid in L-form division is complex. First, our results provided further support for the old idea of ‘nucleoid occlusion’, in which the nucleoid has a localised negative effect on cell division, thereby helping to ensure that progeny cells have intact chromosomes^33,60^. While the recent identification of protein factors, such as Noc in *B. subtilis* and SlmA in *Escherichia coli*, provided the first molecular mechanisms for the nucleoid occlusion effect, it is also clear that in both organisms, the cell division machine is still biased away from the nucleoid in the absence of Noc or SlmA^35,36,61,62^. Importantly, our results clearly show that this bias also occurs for the FtsZ-independent division of L-forms, perhaps pointing to a more fundamental, biophysical basis for the effect. This might plausibly be based on separation of nucleoid and cytoplasmic phases, or a change in the property of the membrane proximal to the nucleoid due to co-transcriptional translation and translocation (transertion) of membrane and secreted proteins.

The second apparent effect of the nucleoid was more surprising. In cell tubes in which replication or segregation was blocked, either spontaneously or by an inhibitor, chains of small anucleate cells were frequently generated by repeated sequential divisions adjacent to a nucleoid area, even in the narrow channels that do not normally support L-form division. This dramatic effect is reminiscent of a biophysical effect called pearling instability, which occurs when the membrane material in cylindrical lipid tubes is subject to tension, e.g., by the action of laser tweezers^21,63,64^, or the budding transition of phospholipid vesicles that leads to the formation of a chain of vesicles at the increase of the area-to-volume ratio^46^. It seems possible that in L-forms these events occur because DNA normally contributes a large proportion of the total mass or effective volume of the cytoplasm. Assuming that synthesis of membrane and cytoplasm (all cell contents other than the nucleoid mass) continue at their normal rates the loss of nucleoid expansion would give an overall increase in A/V, which we have shown previously to drive L-form proliferation^12^. That the pearling events always occurred in regions deficient in or devoid of DNA provides further strong evidence that nucleoids inhibit division. It is interesting to note that the possible role of DNA in cell volume regulation has been highlighted recently by experiments analysing the inflation of the *B. subtilis* forespore compartment by DNA import^65^.

Finally, our findings have implications for attempts to use L-form like division in the development of artificial cells. As well as providing further insights into the key parameters that need to be controlled to drive division, they also suggest that rates of DNA, cytoplasm and membrane synthesis all need to be properly balanced to efficiently coordinate division and chromosome segregation.

## Methods

### Bacterial strains, plasmids and growth conditions

The bacterial strains and plasmid constructs used in this study are shown in Supplementary Table 1. *B. subtilis* transformation was performed by the two-step starvation procedure modified from Anagnostopoulos and Spizizen^66^. Briefly, the strain to be transformed was grown in 3 ml pre-transformation medium (10 ml SMM salts, 250 μl 40% glucose, 100 μl Solution P, 200 μl 20% w/v Casamino acids, 100 μl 2 mg ml^−1^ Tryptophan) with shaking at 37 °C until the optical density (A600 nm) reached 3–3.5. Cells should now be competent. Next, 100 μl competent cells was added into a test tube containing 1 ml pre-warmed transformation medium (10 ml SMM salts, 0.15 ml 40% glucose, 0.05 ml 1 M MgSO_4_, 5 ml 20% w/v Casamino acids, 100 μl 2 mg ml^−1^ Tryptophan), along with the DNA to be transformed (less than 100 μl DNA). The mixture was incubated at 37 °C with shaking for 60–90 min, then plated onto selective plates. SMM contains 0.2% (w/v) (NH4)_2_SO_4_, 1.4% (w/v) K_2_HPO_4_, 0.6% (w/v) KH_2_PO_4_, 0.1% C_6_H_5_Na_3_O_7_·2H_2_O, 0.02% (w/v) MgSO_4_. Solution P contains 357 mM MgSO_4_, 7.14 mM CaCl_2_ and 0.00157% MnSO_4_·4H_2_O.

Walled *B. subtilis* cells were grown on nutrient agar (NA, Oxoid) or in Luria–Bertani broth (LB). *B. subtilis* L-forms were grown in osmoprotective liquid medium NB/MSM at 30 °C without shaking. The NB/MSM medium is composed of 2× magnesium–sucrose–maleic acid (MSM) pH7 (40 mM MgCl_2_, 1 M sucrose, and 40 mM maleic acid) mixed 1:1 with 2× NB (nutrient broth). 0.8% xylose and 0.8 mM IPTG (Isopropyl β-d-1-thiogalactopyranoside) were added as needed. When necessary, antibiotics were added to media at the following concentrations: 5 µg ml^−1^ chloramphenicol, 5 µg ml^−1^ kanamycin; 55 µg ml^−1^ spectinomycin; 12 µg ml^−1^ tetracycline; 1 µg ml^−1^ erythromycin; 25 µg ml^−1^ lincomycin and 200 μg ml^−1^ Penicillin G. L-form strains derived from LR2 were maintained in the L-form state by the addition of Penicillin G and omission of xylose in the growth medium, while those derived from RM121 were stable L-forms and did not require the addition of Penicillin G.

### Protoplast and L-form preparation in liquid medium

Exponentially growing *B*. *subtilis* walled cells (OD600nm of 0.2–0.3) in LB medium with appropriate supplements were harvested and washed once in LB, then resuspended in NB/MSM containing lysozyme (2 mg ml^−1^). The cells were incubated at 37 °C with gentle shaking for 1 h, or until all the rod-shaped cells have been protoplasted. The protoplasts were then diluted (1 in 5000) in NB/MSM containing 200 μg ml^−1^ PenG, and grown at 30 °C without shaking for 2–4 days, during which time the protoplasts would transit into L-forms. The freshly generated L-forms were diluted at least twice in the same medium and cultured at 30 °C, before being used for further experiments.

### Microfluidic system and microscopy

Microfluidic experiments were carried out using a device produced in-house based on that described by Moffitt et al.^29,30^ (Supplementary Fig. 1). Each microfluidic design (chip) contains a set of 3 tracks of different widths, repeated and grouped into 15 μm × 20 μm blocks divided by gutters. The channel widths for the agarose microfluidic chips used are: Chip No. 2 (previously: 0.8, 0.9 and 1.0 µm; new: 0.6, 0.7 and 0.8 µm); Chip No. 6 (1, 1.2 and 1.4 µm); Chip No. 7 (1.8, 2.0 and 2.2 µm) and Chip No. 33 (0.6, 0.7 and 0.8 µm interrupted by diamond shapes). The diamond shapes in Chip No. 33 were spaced 20 μm apart (measured from centre to centre) and measured 3.4 μm at the widest point. The designs were initially created on l-edit software and transferred onto a silicon wafer using lithography and deep reactive-ion etching (DRIE), followed by backfilling with a coating of Tetraethyl orthosilicate oxide (TEOS oxide) to produce channels of the desired dimensions (Lionex Ltd.). Using TEOS oxide backfill negates the need to use expensive e-beam lithography. Replica moulding of the silicon wafer with hard PDMS was used to create the intermediate mould used to transfer the pattern onto the agarose. Patterned agarose pads, with channels ~1.6 μm high, were cast using an intermediate PDMS mould and an ‘agarose casting mould’ made of PDMS, with 4% low melting point agarose (SeaPlague GTG Agarose from Lonsza, gelling temp 26–30 °C) in growth medium containing 1× MSM and 1/5× NB, which then set slowly at 30 °C for 1–2 h.

The structural part of the device, the PDMS chamber block, was cast using a custom designed and milled aluminium mould that matches the size of the mould for casting the agarose pad. Plasma-bonding of the PDMS chamber block to a long cover glass (Agar Scientific Ltd., L4239-2, Coverglass 35 × 64 mm No. 1.5) created a sample chamber. The cover glass which formed the bottom of the sample chamber was coated with BSA (0.5 mg ml^−1^) and allowed to dry. Five microlitre of concentrated L-form culture was added onto the cover glass in the sample chamber, then the patterned agarose pad placed (patterned side down) onto the cells in the sample chamber, trapping bacterial cells in the channels of the agarose pad. The chamber was then sealed with a plasma-treated cover glass (Agar Acientific Ltd., L46s20-5, coverglass 20 × 20 mm No. 5) to the top of the agarose pad. The assembly was left at 30 °C for 20 min to allow plasma bond to set. The PDMS chamber block also contained two buffer reservoirs on either side of, and connected to, the sample chamber, one for inputting fresh medium and the other as the outlet of the spent medium and bacterial cells that were not confined in the tracks. Modified growth medium with 1/5× strength of NB was supplied continuously through the inlet reservoir from a 50 ml syringe, controlled by a syringe pump at a speed of 1 ml h^−1^ using the WinPump Term software (New Era).

Microscopy was performed on a Nikon Eclipse Ti inverted fluorescence microscope system, fitted with an Apo TIRF objective (Nikon 60×/1.49 Oil), as described previously^13^. All time-lapse experiments were carried out at 32 °C unless otherwise indicated. For experiments with the DNA replication inhibitor HB-EMAu (N3-hydroxybutyl 6-(3′-ethyl-4′-methylanilino) uracil;^42^), freshly growing L-form culture was mixed with the inhibitor at 3 µg ml^−1^ then loaded into the microfluidic devices. Sometimes the mixture was incubated at 30 °C from 30 to 40 min prior to loading. The inhibitor was also added to the flow medium at the same concentration to maintain the inhibition.

Time-lapse microscopy of L-forms growing in liquid medium was performed using ibiTreat, 35 mm sterile glass bottom microwell, on a DeltaVision^®^RT microscope (Applied Precision, Washington, USA) as described by Domínguez-Cuevas et al.^67^. Briefly, 200 µl of L-form cells were placed in the dish and left to stand for 10 min. To adhere the cells to the surface of the glass dish, the dish was centrifuged at 100*g* for 5 min using a Beckman Allegra X-12R centrifuge.

For each time-lapse experiment multiple positions were imaged and all experiments were performed at least twice. Images of nucleoids (HU-mCherry) in Figs. 1b, 3a, b, 4–6 and Supplementary Figs. 2–5 were saturated so that dim features could be observed. Unsaturated images were also presented where appropriate.

### Quantitative image analysis

Movies were prepared for quantitative analysis in the following steps. Some images were registered to correct for drift using FIJI/ ImageJ StackReg^68^. Images were manually rotated such that the microchannels were precisely vertical (FIJI, bicubic interpolation). Images were background subtracted using FIJI paraboloid rolling ball, radius 50. Cells in each agarose channel were then manually quality controlled to exclude channels initially loaded with more than one cell, or cells where overgrowth from adjacent channels obscured the cell growth in that channel. Cropped data from individual quality controlled channels were then exported for quantitative analysis in MATLAB.

Nucleoids from cells in each channel were segmented using Otsu’s method. Spurs/connecting noise pixels were removed using image opening with a disk radius 1. Shape and size parameters for segmented nucleoids were then calculated. Cell size was measured by square root of area rather than area because it is a linear quantity. Nucleoid separation was measured as the distance between the centroids of vertically adjacent nucleoids. Nucleoid angle was measured as the angle between vertically adjacent nucleoids, defined such that the angle between two vertically aligned cells was zero degrees. Nucleoid eccentricity was estimated as *e* = (minor axis length)/(major axis length) of the binary object.

Confidence intervals in Fig. 3g, I were estimated by bootstrapping.

Due to the large size of the dataset (*n* = 17,766 nucleoids) outliers or low frequency extrema were observed in the data. In order to visualise average trends in the data, in Fig. 3f, h and Supplementary Fig. 3d, e, it was necessary to zoom in to less than the full data range. The full extent of the data are shown in Supplementary Fig. 6b–e.

Doubling time was determined as the time taken for the length of the cell to double, based on bright-field time-lapse images in the microfluidic devices. Cells were firstly detected and outlined using MicrobeTracker^69^, and then doubling time was calculated using MATLAB as described in Lee et al.^70^. Only single cells that were in the channels not occupied by other cells at the beginning of the experiment were analysed, for the period when they were growing exponentially. The numbers of cells that were tracked and analysed are as the following: for walled cells, 800 nm channels: 70 single cells (monitored for a period of 105, 100 and 130 min, respectively); 900 nm channels: 75 cells (monitored for 120, 115 and 125 min); and 68 cells from the 1000 nm channels (for 105, 110 and 110 min). For L-forms, 179 single cells in the 800 nm channels (monitored for a period of 350, 290 and 240 min, respectively); 199 cells in the 900 nm channels (monitored for 300, 320 and 360 min); and 218 cells from the 1000 nm channels (monitored for 405, 340 and 330 min). R800 (*n* = 70), R900 (*n* = 75), R1000 (*n* = 68), L800 (*n* = 179), R900 (*n* = 199) and R1000 (*n* = 218).

### Reporting summary

Further information on research design is available in the Nature Research Reporting Summary linked to this article.

## Supplementary information

Supplementary Information

Description of Additional Supplementary Files

Supplementary Movie 1

Supplementary Movie 2

Supplementary Movie 3

Supplementary Movie 4

Supplementary Movie 5

Supplementary Movie 6

Supplementary Movie 7

Supplementary Movie 8

Supplementary Movie 9

Supplementary Movie 10

Supplementary Movie 11

Supplementary Movie 12

Supplementary Movie 13

Reporting Summary

## Data Availability

Data supporting the findings of this study are available within the article and Supplementary Information, and are available from the corresponding authors upon request. Source data are provided with this paper.

## Source data

Source Data
