# Supplementary material for: Geometric principles underlying the proliferation of a model cell system
Source: Nat Commun. 2020 Aug 18;11:4149. doi: 10.1038/s41467-020-17988-7 (PMC7434903; doi:10.1038/s41467-020-17988-7)
Supplement: Supplementary file 1 — Supplementary Information [file 41467_2020_17988_MOESM1_ESM.pdf]

Geometric principles underlying the proliferation of a model cell system (Wu *et al.*)

Supplementary Information

Supplementary Figure 1 (Wu *et al.*)

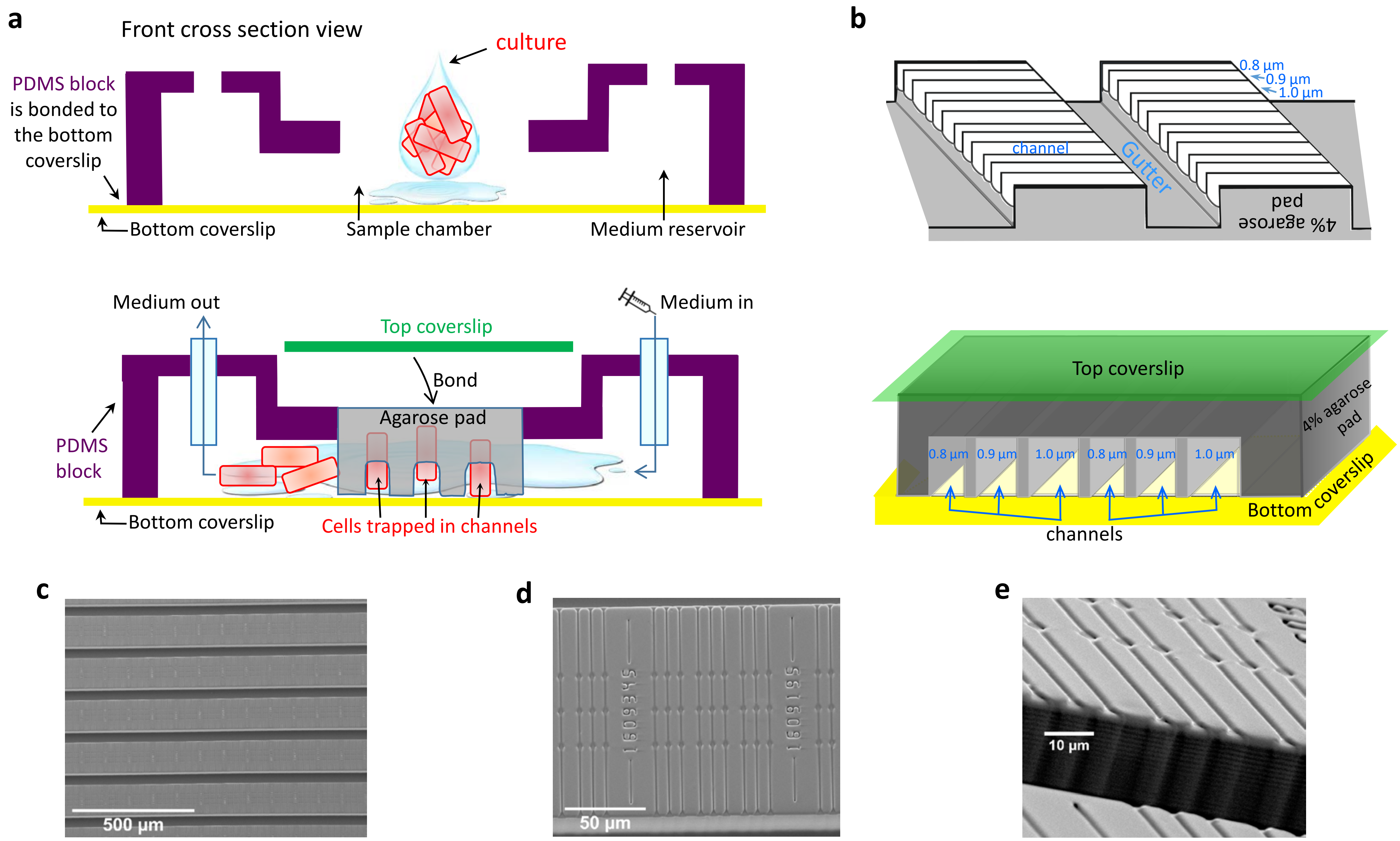

Supplementary Figure 1 The agarose-based microfluidic device adapted from that of Moffitt et al (2012). Related to Figures 2 to 6.

a Schematic diagrams of the microfluidic system and agarose chips (not to scale). **a** Top: Front cross section view of a partially assembled device. Bonding of the PDMS block to the bottom cover glass creates a chamber ready for cells and the printed agarose pad. The chamber is connected to the reservoirs on the two sides. Bottom: An additional cover glass is laid over the agarose pad, and compresses and seals the device. Cells are confined between the patterned agarose pad and the glass bottom coverslip. Medium flows pass both ends of the channels and removes cells as they emerge from the channels. **b** Top panel: a section of an agarose chip shown here upside-down before being bonded to a cover glass. Each chip consists of sections of tracks. Each section of tracks is ~100x100 µm, containing repeats of a set of three tracks of slightly different widths (0.8, 0.9 and 1.0 µm, for example) and are grouped into 15µm x 20µm blocks divided by gutters. After being mounted onto a cover glass (bottom panel), the glass forms the bottom of the channel. The channel has agarose as the sides and the top, and is open on one end or both ends to the gutter.

c-**e** SEM images of the surface pattern that is replica moulded onto the agarose pad using the intermediate mould. As agarose cannot be easily imaged with SEM, PDMS was moulded against the intermediate and cured to check the dimensions and structure of the surface pattern. **c** A zoomed out view to show the repeating pattern of the modules, each individually numbered so locations can be monitored. The darker stripes running left to right are the gutters, through which medium flows. The gutters are 40 µm wide. **b** The repeating unit, this image shows Chip No.33. **e** A tilted view of Chip No.33 to highlight the depth of the channel features (1.6 µm) compared to that of the gutters (40 µm).

Supplementary Figure 2 (Wu *et al.*)

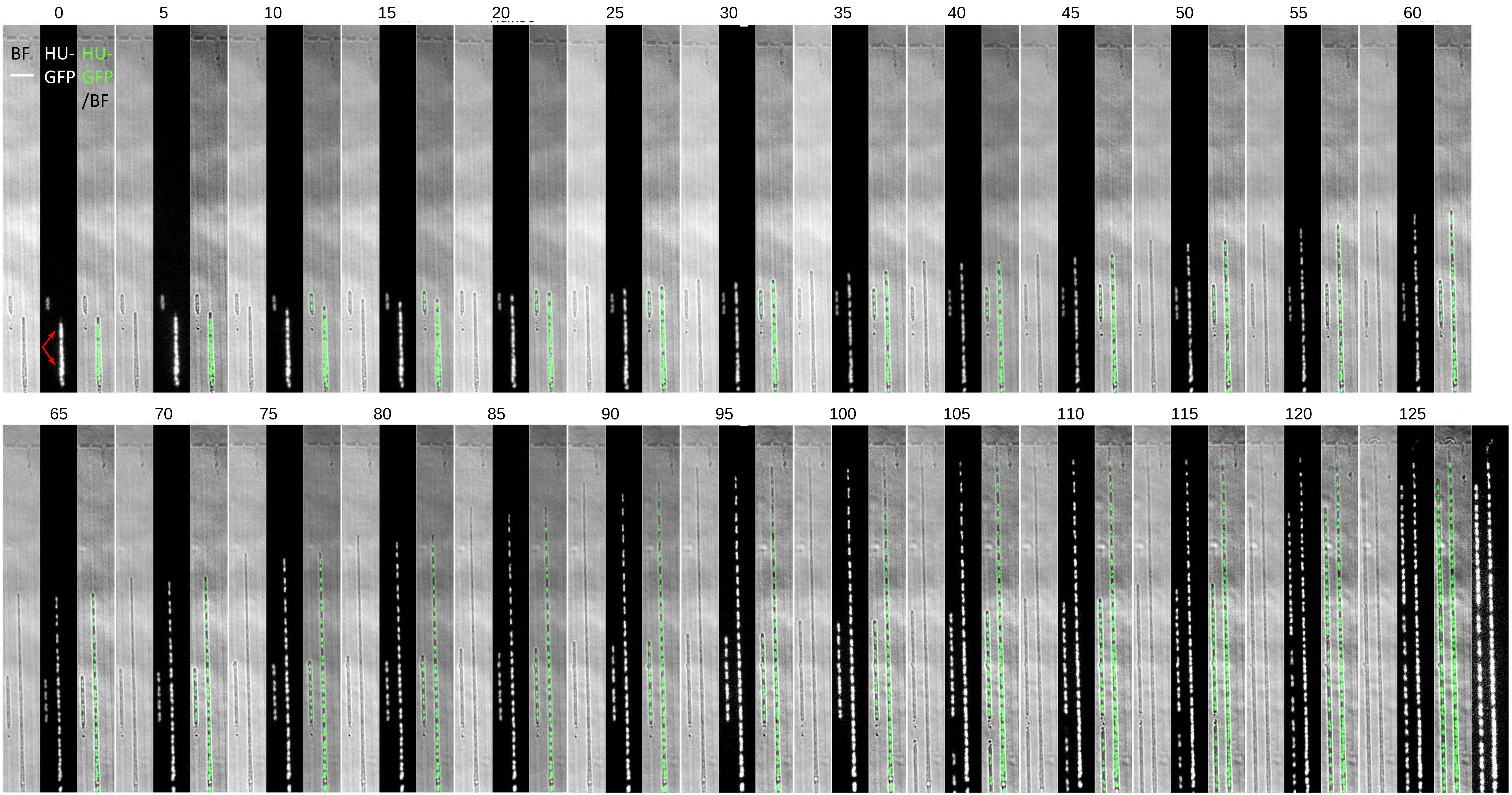

Supplementary Figure 2: Regular chromosome segregation in narrow microfluidic channels. Related to Figure 2a, 3a and 3c.

Full set of still images of a time-lapse experiment presented in Figure 2a and 3a. L-form cells of strain 4739 (LR2 *ΩamyE*::*neo hbsU-gfp*) were loaded into microfluidic chamber (Chip No. 2; channel widths 0.8, 0.9 and 1.0 µm) and grown at 32° C. Images were captured every 5 min. For each frame shown a set of 3 images are presented: a bright field images on the left, a HU-GFP image showing the nucleoids in the middle and the merge on the left (DNA in green). For the last time frame (125 min) an extra image of the nucleoids, with increased brightness to show the DNA in cells exiting the channel, is shown to the right. The small cell on the left was in a 0.9 µm channel; the large cell on the right was in a 0.8 µm channel Arrows: un-resolved chromosomal mass. Scale bar, 5 µm. The experiment was performed more than three times independently, and multiple positions were imaged in each experiment, with similar results.

Supplementary Figure 3 (Wu *et al.*)

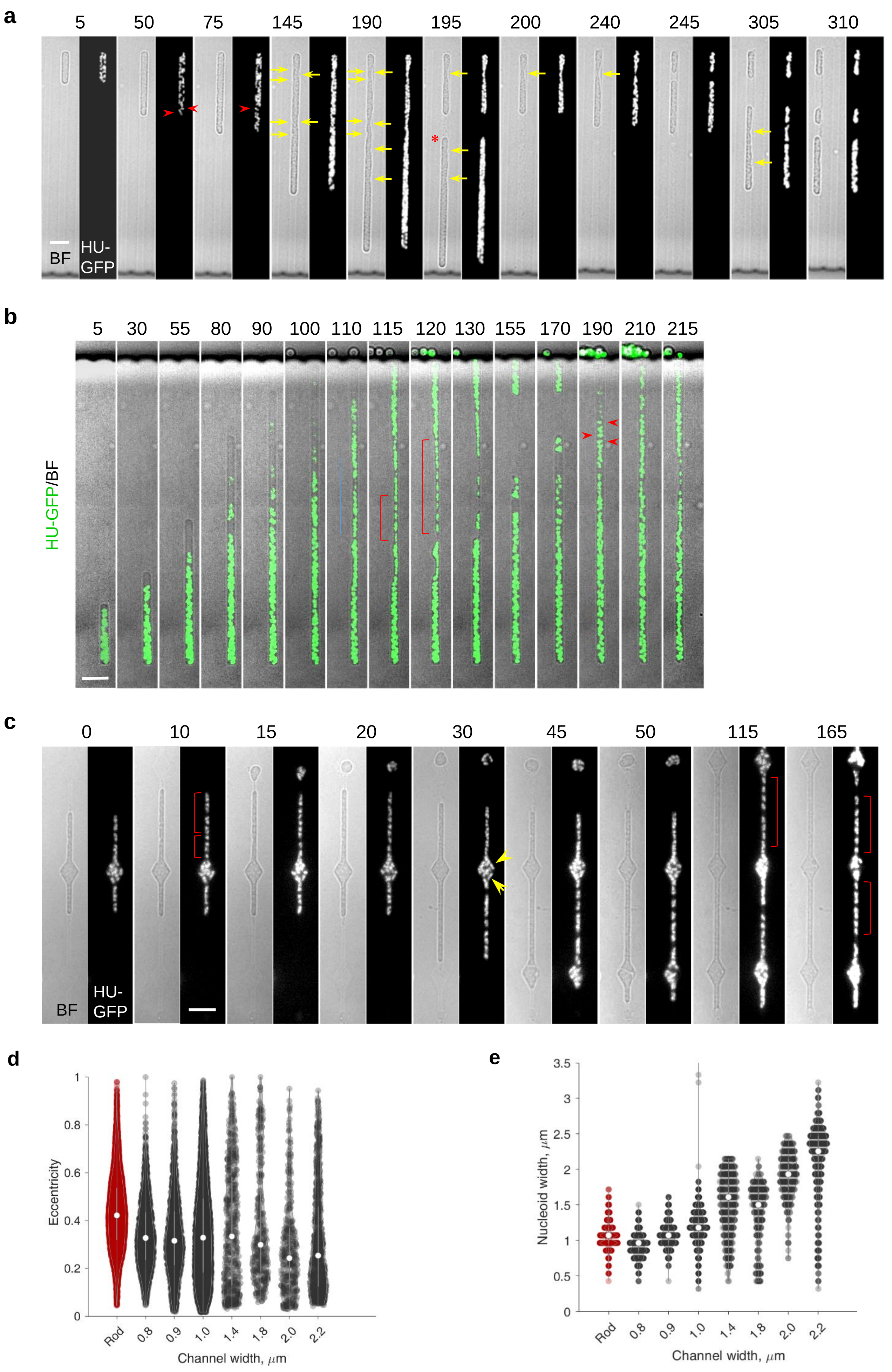

Supplementary Figure 3: L-form division and chromosome distribution in microfluidic channels of various widths. Related to Figures 2 & 3.

a In wide channels cell division occurred more frequently. However, chromosomes are not well separated and are distributed irregularly. These are selected still frames from Supplementary Movie 3. Each time frame shows bright field images on the left and chromosomal DNA labelled with HU-GFP on the right. The cell shown was in a 2.0 µm wide channel (Chip No. 7). Red arrowheads: chromosomes lying horizontally or perpendicularly. Yellow arrows point to regions of membrane constrictions. The daughter cell that escaped from the channel is marked with a red star. Strain: 4739 (LR2 *ΩamyE*::*neo hbsU-gfp*).

b Chromosomes became separated and more regularly distributed in the narrow parts of the wide cell (red brackets) where membrane constrictions persisted. The images are selected still frames from Supplementary Movie 5, shown as the merge of the GFP image (green) and the bright field image (grey). The cell shown was in a 1.4 µm wide channel (Chip No. 6). Strain: 4739 (LR2 *ΩamyE*::*neo hbsU-gfp*).

c In Chip No.33 which contained alternating narrow channels and diamond shapes, dis-organised chromosomes in the diamond parts became regularly distributed in the straight and narrow channels. These are selected still frames from Supplementary Movie 6. Each time frame shows bright field images on the left and chromosomal DNA labelled with HU-GFP on the right. The channel width for the cell shown was 700 nm. Strain: 4741 (LR2 *ΩamyE*::*neo hbsU-gfp aprE::*P_{rpsD}*-mCherry spc)*. Yellow arrowheads indicates two nucleoids in different orientations in the diamond region.

Brackets in **b** & **c** indicate regions where chromosomes appear regularly distributed. Scale bars, 5 µm. All experiment were performed at least twice independently, and multiple positions were imaged in each experiment, with similar results.

d & **e**: Nucleoid eccentricity (**d**) and width (**e**) for walled cells (red; Rod) and L-forms (black) in different channel widths. Violin plots: Circles indicate median, bars indicate upper and lower quartile. Scatter plots: Circles indicate median, error bars indicated 95 % confidence interval from bootstrapping. *n=17766* time-lapse observations of nucleoids descended from 45 mother cells in separate agarose channels. Source data are provided as a Source Data file.

Supplementary Figure 4 (Wu *et al.*)

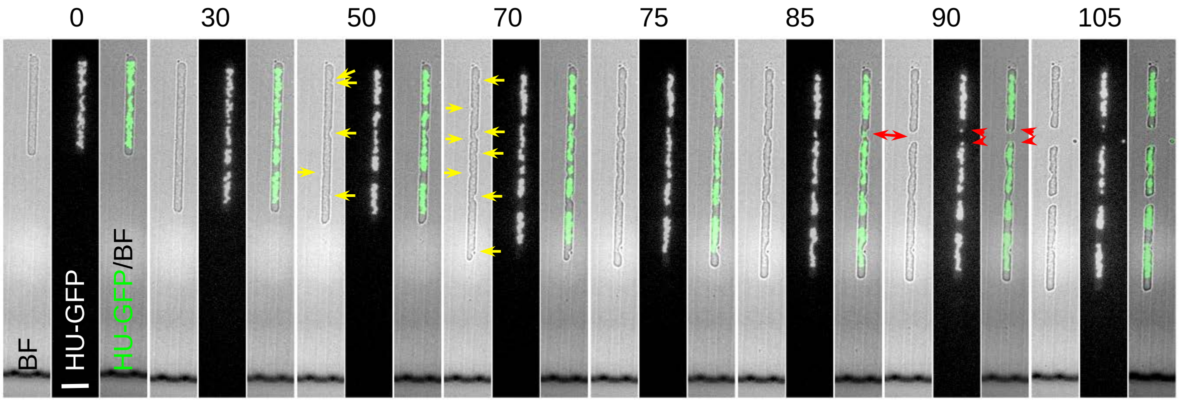

Supplementary Figure 4: Bisection of chromosomes occurs occasionally in L-forms. Related to Figure 4.

A chromosome appeared to have been bisected by division, with two small lobes of DNA (arrowheads in Frame 90 min) retained at the extreme ends of the cell where division has occurred (red arrows in Frames 85 and 90 min). These are selected still frames from Supplementary Movie 8. The cell shown was in a 1.8 µm wide channel (Chip No. 7). Each time frame shows bright field images on the left, chromosomal DNA labelled with HU-GFP in the middle, and a merge of the two on the right (GFP in green). Yellow arrows point to regions of membrane constrictions. Strain: 4739 (LR2 *ΩamyE*::*neo hbsU-gfp).* Scale bar, 5 µm. Similar events could be observed occasionally at different locations from independent experiments (repeated at least twice).

Supplementary Figure 5 (Wu *et al.*)

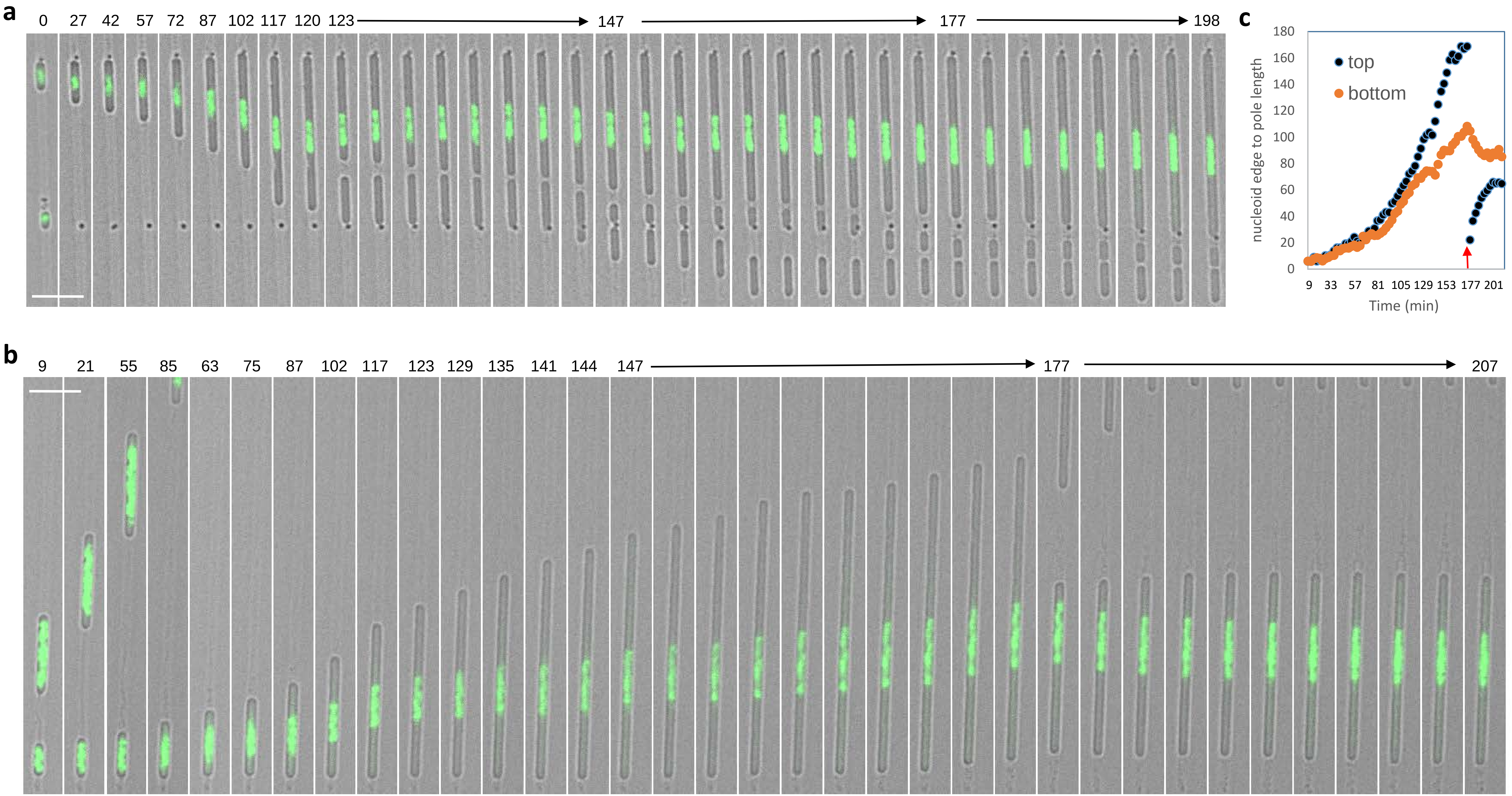

Supplementary Figure 5: Re-centring of the single nucleoid in cells inhibited for DNA replication. Related to Figure 6.

a A fuller set of still images of a time-lapse experiment presented in Figure 6. The merge of the bright field image (grey) and the green fluorescence image of the chromosomal DNA labelled with HU-GFP (green) is shown. After division at 123 min the nucleoid moved towards the distal pole to re-centre itself. Scale bar, 5 µm.

b, **c** Another example of the single nucleoid re-centring after division (at 177 min).

L-forms of strain 4739 (LR2 *ΩamyE*::*neo hbsU-gfp)* were grown in the presence of the DNA replication inhibitor HB-EmAu in liquid culture and after introduction into a microfluidic device. Scale bars, 5 µm. Such events, which occurred in similar experiments, could be clearly observed only in large cells that had a single nucleoid and divided near the nucleoid, and had a sufficiently long time gap before the second division occurred.

Supplementary Figure 6 (Wu *et al.*)

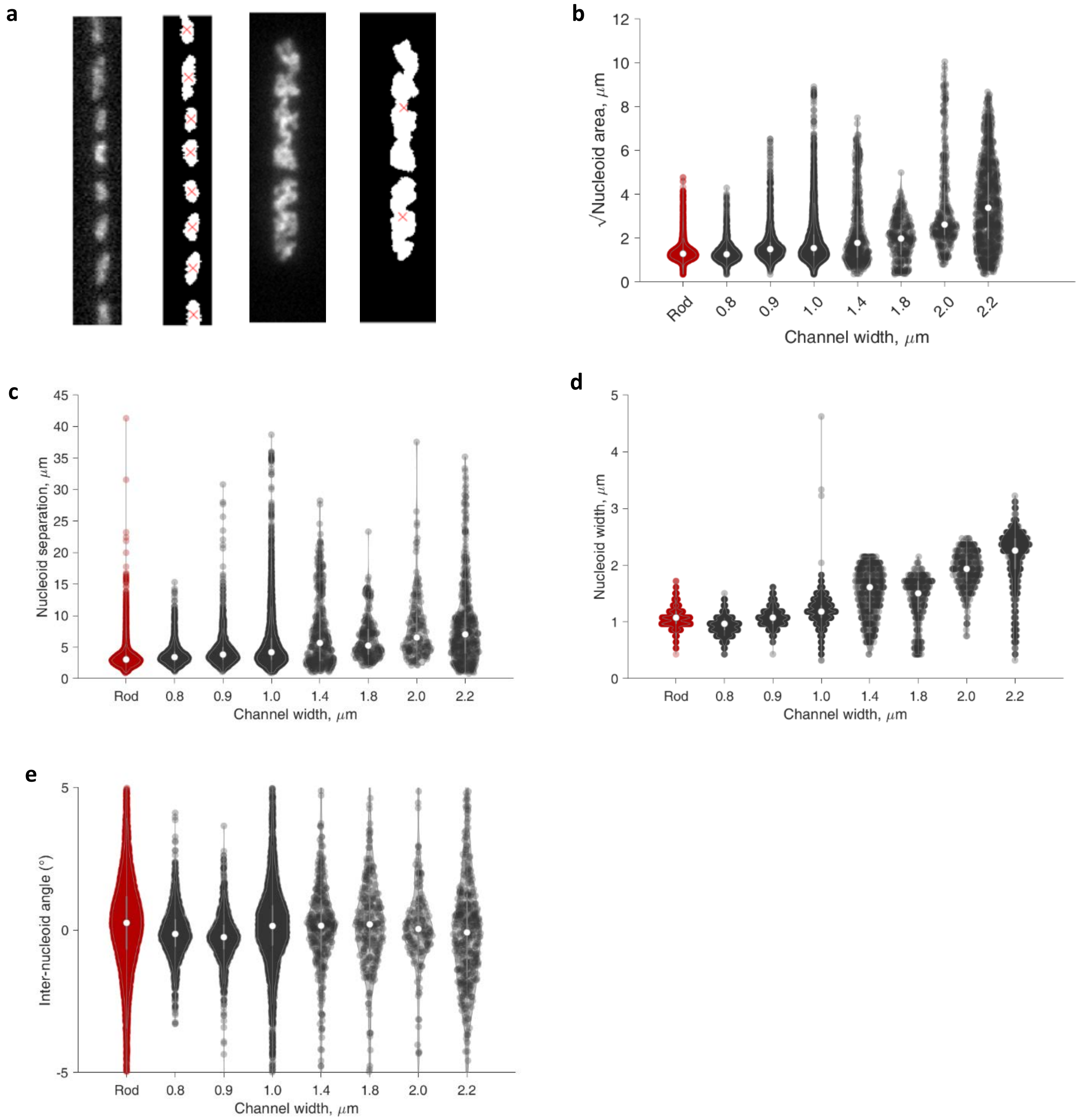

Supplementary Figure 6: Further quantitative analysis of nucleoid shape.

a Exemplar nucleoid segmentations for walled cells (left) and L-form cells in a 2.2 µm wide channel (right).

b-d Violin plots showing full data range including outliers and extrema for Figures 3f, h and Supplementary Figure 3d.

e Distribution of inter-nucleoid angle for all cells. Low frequency long tail of angles greater than 5 degrees not shown in order to visualize average trends.

Source data are provided as a Source Data file.

Supplementary Table 1. Bacterial strains and plasmid used in this study

**Strain**	**Relevant genotype**[a]	**Construction, Source, or Reference**[b]
168CA	*trpC2*	Lab stock
LR2	168ca Ω*spoVD*::*cat* P_{xyl}-*murE* Ω*amyE*::*xylR tet xseB** (Frameshift 22T)	(Mercier et al., 2013)
RM121	168CA *Δ18::tet* pLOSS-P_{spac}-*murC erm*	(Mercier et al., 2013)
PL10	*trpC2 dnaA::*pMUTIN4 *(dnaA'-lacZ ermC* P_{spac}-*dnaA)*	Prolysis Ltd.
SL004	*trpC2 ΩamyE::(cat hbsU-gfp)*	JW Veening & J Errington (unpublished)
Bs138	*trpC2 ΩamyE*::(*cat xylR neo hbsU-gfp*)	(Leaver and Errington, 2005)
1048	*rpoC-gfp cat* P_{1048} *-'rpoC trpC2*	(Lewis et al., 2000)
2010	*trpC2 xylR::tet*	Lab stock
4738	*LR2 aprE::*P_{rpsD}*-mCherry spc*	(Kawai et al., 2014)
4739	LR2 *ΩamyE*::*neo hbsU-gfp*	BS138 → LR2 (kan)
4740	LR2 *ΩamyE*::*neo hbsU-gfp dnaA::*pMUTIN4 *(dnaA'-lacZ ermC Pspac-dnaA)*	PL10 → 4739 (erm/lin)
4741	LR2 *ΩamyE*::*neo hbsU-gfp aprE::*P_{rpsD}*-mCherry spc*	4738 → 4739 (spc)
4742	LR2 *ΩamyE*::*neo hbsU-gfp aprE::*P_{rpsD}*-mCherry spc xylR::tet*	2010 → 4741 (tet)
168ca hbsU-mCherry cat	168ca *ΩamyE*::*cat hbsU-mCherry*	JW Veening & J Errington (unpublished)
4743	168ca *ΩamyE*::(*Ωcat::neo) hbsU-mCherry*	pCm::Nm → 168ca hbsU-mCherry cat (neo; cat^-)
4744	RM121 *ΩamyE*::(*Ωcat::neo) hbsU-mCherry*	4743 → RM121 (neo)
4745	RM121 *ΩamyE*::(*Ωcat::neo) hbsU-mCherry rpoC-gfp cat* P_{xyl}*-'rpoC*	1048 → 4744 (cat)

a: *Ω*, insertion at the indicated locus. The gene or genes inserted are indicated after the double colon. *cat*, chloramphenicol acetyl transferase gene, conferring resistance to chloramphenicol. *neo*, conferring resistance to kanamycin. *phl*, conferring resistance to phleomycin. *spc*, conferring resistance to spectinomycin. P_{xyl} fusion to a xylose-inducible promoter. P_{spac} fusion to an IPTG-inducible promoter.

b: for strains constructed in this work by transformation, the plasmid or the donor strain of the chromosomal DNA is in front of the arrow and the recipient strain is behind the arrow. The antibiotic selection for the transformation in indicated in brackets following the recipient strain.
