# Supplementary material for: Geometric principles underlying the proliferation of a model cell system
Source: Nat Commun. 2020 Aug 18;11:4149. doi: 10.1038/s41467-020-17988-7 (PMC7434903; doi:10.1038/s41467-020-17988-7)
Supplement: Supplementary file 2 — Description of Additional Supplementary Files [file 41467_2020_17988_MOESM2_ESM.pdf]

Description of Additional Supplementary Files

File Name: Supplementary Movie 1
Description: L-forms grow out of focus when unstrained in liquid medium. Related to Figure 1A. A time-lapse experiment showing growth and chromosomes of L-form *B. subtilis* when growing unstrained in liquid medium in a glass-bottomed dish at 30°C. Chromosomes (green, merged with bright field images) soon became difficult to observe as the cells grew and divided in multiple directions. Strain: 4740 (LR2 *Pspac-dnaA ΩamyE*::*neo hbsU-gfp*). Phase contrast and the corresponding GFP images, which were overlaid, were acquired automatically every 5 min. Scale bar, 5 µm.

File Name: Supplementary Movie 2
Description: Some un-constrained L-form cells growing in the gutters of microfluidic systems remain in good focus. Related to Figure 1B. Time-lapse series with an agarose-based microfluidic system showing the growth of L-forms of strain 4745 (RM121 *ΩamyE*::*hbsU-mCherry rpoC-gfp)* in the gutter, from which the panels in Figure 1B were obtained. Phase contrast (left panel) and the corresponding HU-GFP (middle panel) images were acquired automatically every 5 min. Overlay of the phase contrast (grey) and the corresponding HU-GFP (red) images is shown on the right. Scale bar, 5 µm.

File Name: Supplementary Movie 3
Description: L-form division and chromosome distribution in wide microfluidic channels (I). Related to Figure 3B and S3A. A time-lapse series showing cell division events and escaping of daughter cells of L-forms growing in wide channels. Chromosomes are not well separated and are distributed irregularly. The cell shown was in a 2.0 µm wide channel (Chip No. 7). Bright field images are shown on the left and the corresponding HU-GFP images showing the nucleoids are on the right. Strain: 4739 (LR2 *ΩamyE*::*neo hbsU-gfp*). 5 min between frames. Scale bar, 5 µm.

File Name: Supplementary Movie 4
Description: Another example of regular chromosome segregation in microfluidic channels. Time-lapse Series showing L-form cells of strain 4739 (LR2 *ΩamyE*::*neo hbsU-gfp*) growing in microfluidic channels (Chip No. 2; channel widths 0.8, 0.9 and 1.0 µm) at 32°C. Chromosomes can be seen segregating relatively regularly as the cells grew. Images were captured every 5 min. Bright field images are shown on the left and the corresponding HU-GFP images showing the nucleoids are on the right. Related to Figure 3A. Scale bar, 5 µm.

File Name: Supplementary Movie 5
Description: L-form division and chromosome distribution in wide microfluidic channels (II). Related to Figure S3B. A time-lapse series of merged HU-GFP (green) and bright field (grey scale) images showing irregularly distributed chromosomes in L-forms growing in wide channels becoming separated and more regularly distributed in the narrow parts of the cell where membrane constrictions persisted. The cell shown was in a 1.4 µm wide channel (Chip No. 6). Strain: 4739 (LR2 *ΩamyE*::*neo hbsU-gfp*). 5 min between frames. Scale bar, 5 µm.

File Name: Supplementary Movie 6
Description: L-form division and chromosome distribution in mixed shaped microfluidic device. Related to Figure 3E & S3C. A time-lapse series of bright field (left) and HU-GFP (right) images showing chromosome distribution in Chip No.33, which contained alternating narrow channels and diamond shapes. Disorganised chromosomes in the diamond parts became regularly distributed in the straight and narrow channels. Strain: 4741 (LR2 *ΩamyE*::*neo hbsU-gfp aprE*::P_{rpsD}*-mcherry spc)*. 5 min per frame. Scale bar, 5 µm.

File Name: Supplementary Movie 7
Description: Bisection of chromosomes in L-forms growing in wide channels. Related to Figure 4. Chromosomes (green, overlaid with the bright field images shown in grey scale) can be seen passing through areas of invagination, probably prevented division. A small cell with little amount of DNA (Frames 27 onwards) appeared not growing, probably because its chromosome is incomplete. The cell shown was in a 1.8 µm wide channel (Chip No. 7). 5 min between frames. Strain: 4739 (LR2 *ΩamyE*::*neo hbsU-gfp).* Scale bar, 5 µm.

File Name: Supplementary Movie 8
Description: Another example of chromosome bisection in L-forms growing in wide channels. Related to Figure S4. A chromosome (green, overlaid with the bright field images shown in grey scale) appeared to have been bisected by division, generating two small lobes of DNA (Frame 19) that retained at the extreme ends of the cell where division had occurred. The cell shown was in a 1.8 µm wide channel (Chip No. 7). Strain: 4739 (LR2 *ΩamyE*::*neo hbsU-gfp).* Scale bar, 5 µm.

File Name: Supplementary Movie 9
Description: DNA-less 'beads' produced by L-form in narrow channels under normal growth conditions. Related to Figure 5A. The time-lapse series shows cells in the gutter grew into the narrow channels with the mass of the nucleoid excluded from entry, generating strings of DNA-less beads in narrow channels. Each frame shows chromosomes in green overlaid with the corresponding bright field image. Strain: 4739 (LR2 *ΩamyE*::*neo hbsU-gfp).* 5 min between frames. Scale bar, 5 µm.

File Name: Supplementary Movie 10
Description: Small DNA-free cells generated by divisions in DNA-free region of cells in wide channels. Related to Figure 5C. A cell, appeared to be defective in chromosome replication / segregation (for unknown reason), produced many small DNA-free daughter cells of various sizes. The cell shown was in a 2.2 µm wide channel (Chip No. 7). Each frame shows bright field images on the left and chromosomal DNA labelled with HU-mCherry on the right. 5 min between frames. Strain: 4739 (LR2 *ΩamyE*::*neo hbsU-gfp).* Scale bar, 5 µm.

File Name: Supplementary Movie 11
Description: DNA-less cells produced in narrow channels by L-forms inhibited for DNA replication. The time-lapse series shows formation of DNA-less 'beads on a string'. The replication inhibitor HB-EmAu was present in the medium throughout the time-lapse experiment. Strain: 4739 (LR2 *ΩamyE*::*neo hbsU-gfp).* 3 min between frames. Related to Figure 5D. Scale bar, 5 µm.

File Name: Supplementary Movie 12

Description: Large DNA-free cells divided into smaller cells. Related to Figure 5E. The time-lapse series shows formation of large DNA-less cells in narrow channels by L-forms inhibited for DNA replication, which divided further into smaller DNA-less daughters. The replication inhibitor HB-EmAu was present in the medium throughout the time lapse experiment. Strain: 4739 (LR2 *ΩamyE*::*neo hbsU-gfp).* 3 min between frames. Scale bar, 5 µm.

File Name: Supplementary Movie 13

Description: Re-centring of the single nucleoid after division. Time lapse of the experiment shown in Figure 6. The bright field image (grey) and the green fluorescence image of the chromosomal DNA labelled with HU-GFP (green) are merged. After division the asymmetrically located nucleoid moved towards the distal pole to re-centre itself. L-forms of strain 4739 (LR2 *ΩamyE*::*neo hbsU-gfp)* were grown in the presence of the DNA replication inhibitor HB-EmAu in liquid culture and after introduction into a microfluidic device. 3 min per frame. Scale bar, 5 µm.
