# Supplementary material for: Geometric principles underlying the proliferation of a model cell system
Source: Nat Commun. 2020 Aug 18;11:4149. doi: 10.1038/s41467-020-17988-7 (PMC7434903; doi:10.1038/s41467-020-17988-7)
Supplement: Supplementary file 17 — Source Data [file 41467_2020_17988_MOESM17_ESM.zip › Wu2020_nucleoid_analysis_raw_data/Wu2020_nucleoidAnalysis_manuscript_figures.html]

Untitled 

Code to plot Wu 2020 Nat Comms nucleoid analysis Figures 3F-I, SI Fig 3D-E, SI Figure 6

Written by Seamus Holden, Newcastle University

Released under the GNU GPL v3 © Seamus Holden

Requires https://github.com/bastibe/Violinplot-Matlab

load("Wu2020\_nucleoidRawData.mat");

plotOrder={'Rod', '0.8{\mu}m', '0.9{\mu}m', '1.0{\mu}m',...

'1.4{\mu}m', '1.8{\mu}m', '2.0{\mu}m', '2.2{\mu}m'};

plotOrderAlt={'Rod', '0.8', '0.9', '1.0',...

'1.4', '1.8', '2.0', '2.2'};

bactWidth = [0.85, 0.8,0.9,1.0,1.4,1.8,2.0,2.2];

bactType = {'rod','lform','lform','lform','lform','lform','lform','lform'}

bactType = 1×8 cell

'rod' 'lform' 'lform' 'lform' 'lform' 'lform' 'lform' 'lform'

bactLabel={'Rod', '0.8{\mu}m', '0.9{\mu}m', '1.0{\mu}m',...

'1.4{\mu}m', '1.8{\mu}m', '2.0{\mu}m', '2.2{\mu}m'};

%stats calculations

rod\_w = bactWidth(1);

rod\_w = 0.8500

rod\_areaObs = sqrt(nucleoidData.Area(strcmp(nucleoidData.Label,'Rod')));

rod\_areaStat = quantile(rod\_areaObs, [0.25, 0.5, 0.75]); %iqr(1), median qr(3)

%have to filter out any nans for bootstrapping

bootdata = rod\_areaObs(~isnan(rod\_areaObs));

rod\_area95ci = [bootci(2000,{@median,bootdata})]';

rod\_distObs = sqrt(nucleoidData.Dist(strcmp(nucleoidData.Label,'Rod')));

rod\_distStat = quantile(rod\_distObs, [0.25, 0.5, 0.75]); %iqr(1), median qr(3)

bootdata = rod\_distObs(~isnan(rod\_distObs));

rod\_dist95ci = [bootci(2000,{@median,bootdata})]';

kk=1;

for ii = 2:numel(bactLabel)

lform\_w(kk,1) = bactWidth(ii);

lform\_areaObs{kk} = sqrt(nucleoidData.Area(strcmp(nucleoidData.Label,bactLabel{ii})));

lform\_areaStat(kk,:) = quantile(lform\_areaObs{kk}, [0.25, 0.5, 0.75]); %iqr(1), median qr(3)

bootdata = lform\_areaObs{kk}(~isnan(lform\_areaObs{kk}));

lform\_area95ci(kk,:) = [bootci(2000,{@median,bootdata})]';

lform\_distObs{kk} = sqrt(nucleoidData.Dist(strcmp(nucleoidData.Label,bactLabel{ii})));

lform\_distStat(kk,:) = quantile(lform\_distObs{kk}, [0.25, 0.5, 0.75]); %iqr(1), median qr(3)

bootdata = lform\_distObs{kk}(~isnan(lform\_distObs{kk}));

lform\_dist95ci(kk,:) = [bootci(2000,{@median,bootdata})]';

kk=kk+1;

end

Figure 3F channel width vs sqrt(nucleoid area)

violincolor = {[0.7,0,0],[0.2,0.2,0.2]};

nucleoidData.LabelAlt=nucleoidData.Label;

nucleoidData.LabelAlt=strrep(nucleoidData.LabelAlt,'{\mu}m','');

figure;

hS= violinplot(sqrt(nucleoidData.Area),nucleoidData.LabelAlt,'GroupOrder',plotOrderAlt);

hS(1).ViolinColor = violincolor{1};

for ii = 2:8

hS(ii).ViolinColor = violincolor{2};

end

ylabel('\surd{(Nucleoid area)}, {\mu}m')

ylim([0 8]);

ax=gca;

ax.FontSize=12;

%xtickangle(ax,45)

ax.TickDir='out';

xlabel('Channel width, {\mu}m')

h=gcf;

h.Position(4)=420;

h.Position(3)=1000\*2/3;

Figure 3G channel width vs sqrt(nucleoid area) scatter plot

figure

hold all;

%use 95 % ci of the median for the plotting here

errorbar(rod\_w, rod\_areaStat(2),rod\_areaStat(2)-rod\_area95ci(1),rod\_area95ci(2)-rod\_areaStat(2),'o','Color',[.7 0 0],'LineWidth',2);

errorbar(lform\_w, lform\_areaStat(:,2),lform\_areaStat(:,2)-lform\_area95ci(:,1),lform\_area95ci(:,2)-lform\_areaStat(:,2),'ko','LineWidth',2);

P = polyfit(lform\_w,lform\_areaStat(:,2),1);

yfit = P(1)\*lform\_w+P(2);

plot(lform\_w,yfit,'-','Color',[0 0 0.7],'LineWidth',2);

legend({'Rod','Lform'},'Location','northwest');

xlabel('Channel width, {\mu}m');

ylabel('\surd{(Nucleoid area)}, {\mu}m')

xlim([0.5 2.5])

ylim([1 4]);

ax=gca;

ax.FontSize=12;

ax.TickDir='out';

h=gcf;

h.Position(4)=420;

h.Position(3)=1000\*1/3;

Figure 3H channel width vs nucleoid separation

figure

hS=violinplot(nucleoidData.Dist,nucleoidData.LabelAlt,'GroupOrder',plotOrderAlt);

hS(1).ViolinColor = violincolor{1};

for ii = 2:8

hS(ii).ViolinColor = violincolor{2};

end

ylabel('Nucleoid separation, {\mu}m');

ylim([0 20]);

ax=gca;

ax.FontSize=12;

ax.TickDir='out';

xlabel('Channel width, {\mu}m')

ylabel('Nucleoid separation, {\mu}m');

h=gcf;

h.Position(3)=1000\*2/3;

Figure 3I scatter plot of channel width v nucleoid separation

Plot the medians with CI + linear fit

figure

hold all;

%use 95 % ci of the median for the plotting here

errorbar(rod\_w, rod\_distStat(2),rod\_distStat(2)-rod\_dist95ci(1),rod\_dist95ci(2)-rod\_distStat(2),'o','Color',[.7 0 0],'LineWidth',2);

errorbar(lform\_w, lform\_distStat(:,2),lform\_distStat(:,2)-lform\_dist95ci(:,1),lform\_dist95ci(:,2)-lform\_distStat(:,2),'ko','LineWidth',2);

P = polyfit(lform\_w,lform\_distStat(:,2),1);

yfit = P(1)\*lform\_w+P(2);

plot(lform\_w,yfit,'-','Color',[0 0 0.7],'LineWidth',2);

legend({'Rod','Lform'},'Location','northwest');

xlabel('Channel width, {\mu}m');

ylabel('Nucleoid separation, {\mu}m');

xlim([0.5 2.5])

ylim([1.5 3]);

ax=gca;

ax.FontSize=12;

ax.TickDir='out';

h=gcf;

h.Position(4)=420;

h.Position(3)=1000\*1/3;

SI Figure 3D

figure

hS=violinplot(nucleoidData.Circularity,nucleoidData.LabelAlt,'GroupOrder',plotOrderAlt);

hS(1).ViolinColor = violincolor{1};

for ii = 2:8

hS(ii).ViolinColor = violincolor{2};

end

xlabel('Channel width, {\mu}m')

ylabel('Eccentricity');%Eccentricity and Circularity are synonymous, but eccentricity seems a bit more intuitive description

h=gcf;

h.Position(3)=1000;

ax=gca;

ax.FontSize=12;

ax.TickDir='out';

xtickangle(ax,45)

ylim([0 1]);

h=gcf;

h.Position(4)=420;

h.Position(3)=1000\*1/2;

SI Figure 3E

figure;

hS=violinplot(nucleoidData.Width,nucleoidData.LabelAlt,'GroupOrder',plotOrderAlt);

hS(1).ViolinColor = violincolor{1};

for ii = 2:8

hS(ii).ViolinColor = violincolor{2};

end

xlabel('Channel width, {\mu}m')

ylabel('Nucleoid width, {\mu}m')

ylim([0 3.5]);

ax=gca;

ax.FontSize=12;

ax.TickDir='out';

xtickangle(ax,45)

h=gcf;

h.Position(4)=420;

h.Position(3)=1000\*1/2;

SI Figure 6B: Same as Figure 3F but zoomed out fully

figure;

hS= violinplot(sqrt(nucleoidData.Area),nucleoidData.LabelAlt,'GroupOrder',plotOrderAlt);

hS(1).ViolinColor = violincolor{1};

for ii = 2:8

hS(ii).ViolinColor = violincolor{2};

end

ylabel('\surd{Nucleoid area}, {\mu}m')

ax=gca;

ax.FontSize=14;

xtickangle(ax,45)

xlabel('Channel width, {\mu}m')

h=gcf;

h.Position(4)=420;

h.Position(3)=1000\*2/3;

ax.TickDir='out';

SI Figure 6C - same as Figure 3H but zoomed out fully

figure

hS=violinplot(nucleoidData.Dist,nucleoidData.LabelAlt,'GroupOrder',plotOrderAlt);

hS(1).ViolinColor = violincolor{1};

for ii = 2:8

hS(ii).ViolinColor = violincolor{2};

end

ylabel('Nucleoid separation, {\mu}m');

ax=gca;

ax.FontSize=12;

ax.TickDir='out';

xlabel('Channel width, {\mu}m')

ylabel('Nucleoid separation, {\mu}m');

h=gcf;

h.Position(4)=420;

h.Position(3)=1000\*2/3;

SI Figure 6D - same as SI Figure 3D but zoomed out fully

figure;

hS=violinplot(nucleoidData.Width,nucleoidData.LabelAlt,'GroupOrder',plotOrderAlt);

hS(1).ViolinColor = violincolor{1};

for ii = 2:8

hS(ii).ViolinColor = violincolor{2};

end

xlabel('Channel width, {\mu}m')

ylabel('Nucleoid width, {\mu}m')

ax=gca;

ax.FontSize=12;

ax.TickDir='out';

h=gcf;

h.Position(4)=420;

h.Position(3)=1000\*2/3;

SI Figure 6E

channelAngle = 90-nucleoidData.Theta/(pi)\*180;

figure

hS=violinplot(channelAngle,nucleoidData.LabelAlt,'GroupOrder',plotOrderAlt);

hS(1).ViolinColor = violincolor{1};

for ii = 2:8

hS(ii).ViolinColor = violincolor{2};

end

xlabel('Channel width, {\mu}m')

ylabel(['Inter-nucleoid angle (' char(176),')']);

ylim([-5 5])

ax=gca;

ax.FontSize=12;

ax.TickDir='out';

h=gcf;

h.Position(4)=420;

h.Position(3)=1000\*2/3;

  
